# N-cadherin inhibitor creates a microenvironment that protect TILs from immune checkpoints and Treg cells

**DOI:** 10.1136/jitc-2020-002138

**Published:** 2021-03-10

**Authors:** Yi Sun, Jun Jing, Huan Xu, Lingfan Xu, Hailiang Hu, Cai Tang, Shengzhuo Liu, Qiang Wei, Ruiqi Duan, Ju Guo, Lu Yang

**Affiliations:** 1 Department of Urology, West China Hospital of Sichuan University, Chengdu, China; 2 Department of Pathology, Duke University School of Medicine, Durham, NC, USA; 3 Department of Rheumatology and Clinical Immunology, Sun Yat-Sen Memorial Hospital, Sun Yat-Sen University, Guangzhou, China; 4 Guangdong Provincial Key Laboratory of Malignant Tumor Epigenetics and Gene Regulation, Sun Yat-Sen Memorial Hospital, Sun Yat-Sen University, Guangzhou, China; 5 Department of Urology, Shanghai Changhai Hospital of Second Military Medical University, Shanghai, China; 6 West China School of Public Health and West China Fourth Hospital, Sichuan University, Chengdu, China; 7 Department of Obstetrics and Gynecology/Key Laboratory of Birth Defects and Related Diseases of Women and Children, West China Second Hospital of Sichuan University, Chengdu, China; 8 Department of Urology, The First Affiliated Hospital of Nanchang University, Nanchang, China

**Keywords:** immune reconstitution, prostatic neoplasms

## Abstract

**Background:**

Few patients with prostate cancer benefit from current immunotherapies. Therefore, we aimed to explore new strategies to change this paradigm.

**Methods:**

Human tissues, cell lines and in vivo experiments were used to determine whether and how N-cadherin impacts the production of programmed death ligand-1 (PD-L1) and indole amine 2,3-dioxygenase (IDO-1) and whether N-cadherin can increase the production of effector (e)Treg cells. Then, we used PC3-bearing humanized non-obese diabetic/severe combined immunodeficiency IL2Rγnull (hNSG) mice with an intravenous injection of human CD34+ hematopoietic stem cells into the tail vein to evaluate whether the N-cadherin antagonist N-Ac-CHAVC-NH2 (designated ADH-1) could improve the therapeutic effect of tumor-infiltrating lymphocyte (TIL)-related treatment.

**Results:**

N-cadherin dramatically upregulated the expression of PD-L1 and IDO-1 through IFN-γ (interferongamma) signaling and increasing the production of free fatty acids that could promote the generation of eTreg cells. In preclinical experiments, immune reconstitution mediated by TILs slowed tumor growth and extended the survival time; however, this effect disappeared after immune system suppression by PD-L1, IDO-1 and eTreg cells. Furthermore, ADH-1 effectively reduced immunosuppression and enhanced TIL-related therapy.

**Conclusions:**

These data show that the N-cadherin antagonist ADH-1 promotes TIL antitumor responses. This important hurdle must be overcome for tumors to respond to immunotherapy.

## Background

Hormone treatment may induce the conversion of androgen-dependent prostate tumors into androgen-independent tumors, and a large proportion of castration-resistant prostate cancer (CRPC) cases eventually progress to highly aggressive small cell neuroendocrine prostate cancer (SCC) with a poor prognosis.^1–3^ Due to these circumstances, immunotherapy has emerged as a valuable strategy for several tumor types.^4 5^ Because tumor-infiltrating lymphocytes (TILs) are key to unlocking effective cancer immunotherapy,^6–8^ researchers have focused on establishing mouse models to explore TIL-related caner immunotherapy strategies.^9 10^ However, the immune dysfunction mechanism, including programmed death ligand-1 (PD-L1) and indole amine 2,3-dioxygenase (IDO-1), has been shown to induce T lymphocyte anergy and/or apoptosis.^11–13^ Thus, the inhibition of IDO-1 and PD-L1 is hypothesized to contribute to TIL-related tumor immunotherapy. In addition, the epithelial-mesenchymal transition (EMT) occurs in advanced cancers and enhances the immunosuppressive action by increasing the production of PD-L1 and IDO-1.^14–18^ A recent study reported that N-cadherin initiates the EMT process.^19^ Therefore, we hypothesized that N-cadherin deletion can improve the efficacy of TIL-related immunotherapy by reversing the EMT and then decreasing the production of PD-L1 and IDO-1. The ratio of CD8+ T cells to regulatory T (Treg) cells in the tumor microenvironment (TME) is also an important factor for predicting prognosis and clinical efficacies.^20 21^ Furthermore, anti-CTLA-4 may be effective in some patients with lower response rates to programmed death-1 (PD-1) blockade. Therefore, we further explored the impact of N-cadherin on effector Treg (eTreg) cells and provide evidence for the potential contribution of N-cadherin to immunotherapy. To this end, we attempted to provide evidence regarding the use of N-cadherin blocker to improve the efficiency of prostate cancer immunotherapy. Recently, several types of N-cadherin antagonists have been discovered, such as synthetic linear peptides, synthetic cyclic peptides and non-peptidyl peptidomimetics.^22^ Synthetic cyclic peptides harboring the HAV (histidine-alanine-valine) motif were first shown to act as N-cadherin antagonists,^23^ and the most studied cyclic peptide is N-Ac-CHAVC-NH2 (designated ADH-1).^24^ However, use of N-cadherin antagonists in the clinic as oncology therapeutics is at an early stage of investigation, only a few clinical trials have been conducted with ADH-1 for the treatment of ovarian cancer^25^ and melanoma,^26^ and even fewer in prostate cancer. In this study, we used ADH-1 and TIL-related therapy together to treat prostate cancer in a mouse model to explore the significance of ADH-1 as a potential treatment strategy in prostate cancer.

## Results

### Expression of the immunosuppressive factors PD-L1/IDO-1 is associated with N-cadherin expression

**Figure 1:**
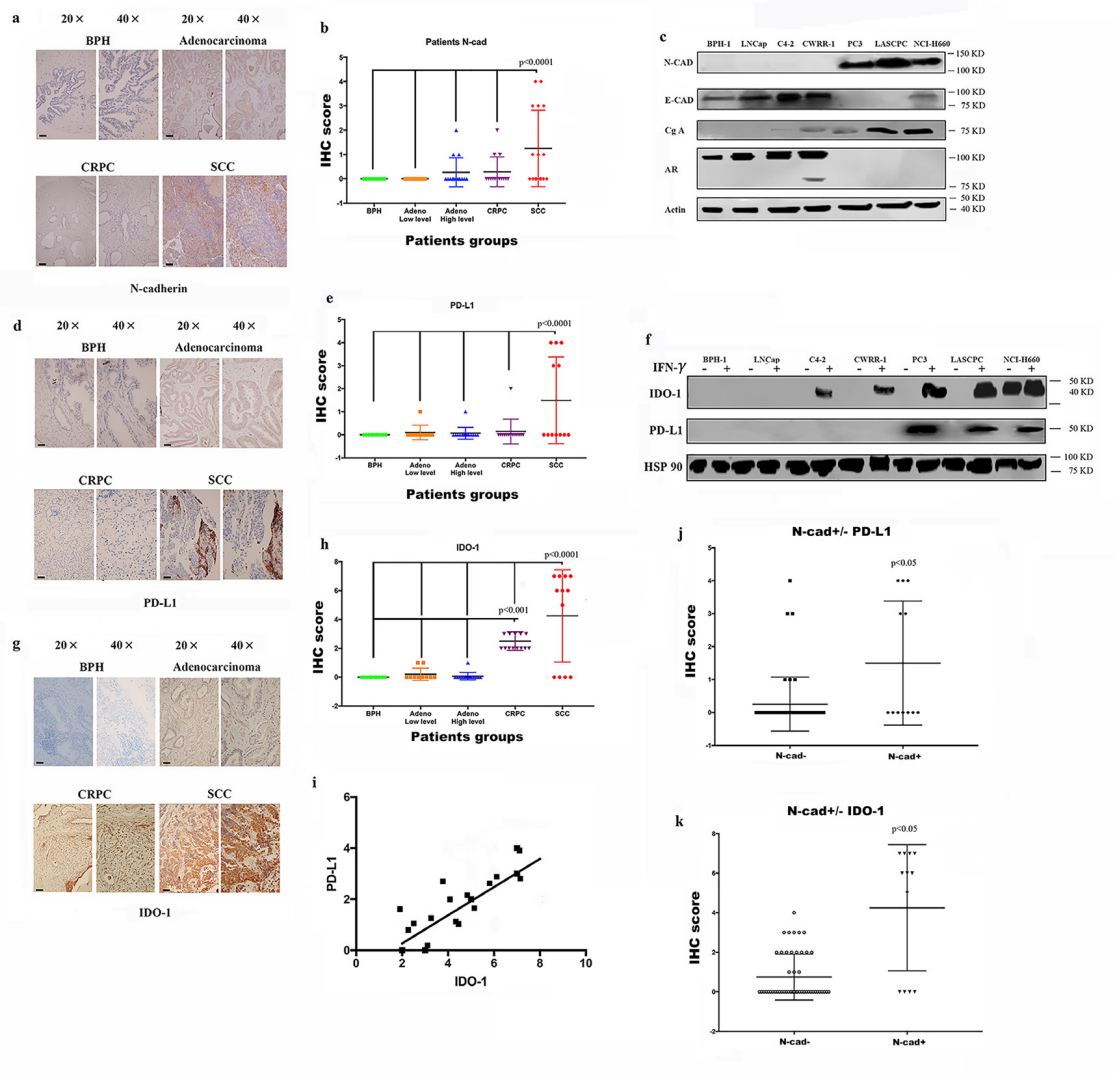
Expression of N-cad, IDO-1 and PD-L1 in patient tissue samples and cell lines. IHC sapmles including 30 benign samples, 15 low level adenocarcinoma, 15 high level adenocarcinoma, 18 CRPC samples and 16 SCC samples. (A) Immunohistochemical staining for N-cad expression in benign, adenocarcinoma (Gleason score greater than 7 was considered high; lower than 7 was low), CRPC and SCC tissue samples, the black bars in the IHC view are 200 µm. (B) IHC scores for N-cad expression in different tumors; only SCC samples had detectable expression. (C) Expression of N-cad, E-cadherin (E-cad) and Chromogranin-A (Cg-A) in different cell lines. (D) IHC staining for PD-L1 in different tumors; the black bars in the IHC view are 200 µm. (E) IHC scores for PD-L1 expression in different tumors; only SCC samples had detectable expression; (F) Expression of PD-L1 in different cell lines. (G) IHC staining for PD-L1 in different tumors; the black bars in the IHC image are 200 µm. (H) IHC scores of different tumors. The SCC group had the highest score, and the CRPC group had the second highest. (I) Analysis of the linear relationship between IDO-1 expression and PD-L1 expression. (J and K) Expression of PD-L1 and IDO-1 in N-cad-positive and N-cad-negative samples. CRPC, castration-resistant prostate cancer; IDO-1, indole amine 2,3-dioxygenase;IFN-γ, interferon gamma; IHC, immunohistochemistry; N-cad, N-cadherin; PD-L1, programmed death ligand-1; SCC, small cell neuroendocrine prostate cancer.

As shown in our previous study, N-cadherin mediates prostate cancer invasion and metastasis by activating EMT,^19^ and some researchers have shown a link between EMT and immunosuppression and an increase in the expression of immunosuppression checkpoints.^14–18^ Therefore, we performed immunohistochemistry (IHC) staining of tissue microarrays (TMAs) containing human prostate cancer samples of different stages using antibodies against N-cadherin and PD-L1/IDO-1 to determine the effect of N-cadherin on the expression of immunosuppressive factors in prostate cancer. Scattered or clustered SCC cells expressing N-cadherin were identified (figure 1A), and according to the IHC score (figure 1B), N-cadherin expression was detected only in SCC tissue samples, but not in benign, adenocarcinoma or CRPC tissue samples. We also performed a cell-based experiment in which we selected different cell lines to represent the different types of prostate cancer. BPH-1 is a benign prostate cell line, and LNCap is an early-stage tumor cell line that usually represents adenocarcinoma. C4-2 and CWRR1 cells are commonly used to represent the CRPC phenotype. PC3, LASCPC-01 and NCI0H660 cells were selected as SCC cell lines. As shown in figure 1C, the cell lines with N-cadherin expression (PC3, LASCPC and NCI-H600) also expressed E-cadherin at low levels. These results are consistent with published data showing that N-cadherin enhances EMT,^19^ and the EMT and neuroendocrine differentiation share similar properties and typically cooccur in advanced cancers.^27–31^ Interestingly, as shown in figure 1D, we confirmed that PD-L1 was observed only in N-cadherin-positive tissues (the SCC group). The analysis of the PD-L1 expression signature is shown in figure 1E, and the IHC score for PD-L1 in the SCC group was substantially higher than the scores in the other groups. When we assessed the expression of PD-L1/IDO-1 in cell lines, PD-L1 was not expressed in any cell line (figure 1F). According to some reports, PD-L1 and IDO-1 are primarily associated with interferongamma (IFN-γ) signaling,^32^ and online supplemental figure 1A confirms that higher IFN-γ levels were expressed in tumor tissues than in benign tissues and that IFN-γ also accumulated in lymphocytes, where it simulated the TME. Therefore, IFN-γ was selected to determine the effects and mechanism by which N-cadherin mediates PD-L1/IDO-1 production. Figure 1F and online supplemental figure 1B, C present the PD-L1 and IDO-1 proteins and messenger RNAs (mRNAs); PD-L1 expression was detected only in the cell lines in which N-cadherin was expressed, and IDO-1 expression was also increased in those cells. In addition, when IDO-1 expression was compared among the different tissue groups according to the IHC results, the highest level of IDO-1 was observed in the N-cadherin-positive group (figure 1G, H). Figure 1I shows the positive correlation between PD-L1 and IDO-1. Figure 1J, K provide additional evidence that PD-L1 and IDO-1 were expressed at higher levels in the N-cadherin-positive group than in the N-cadherin-negative group.

10.1136/jitc-2020-002138.supp4 Supplementary data

**Figure 5:**
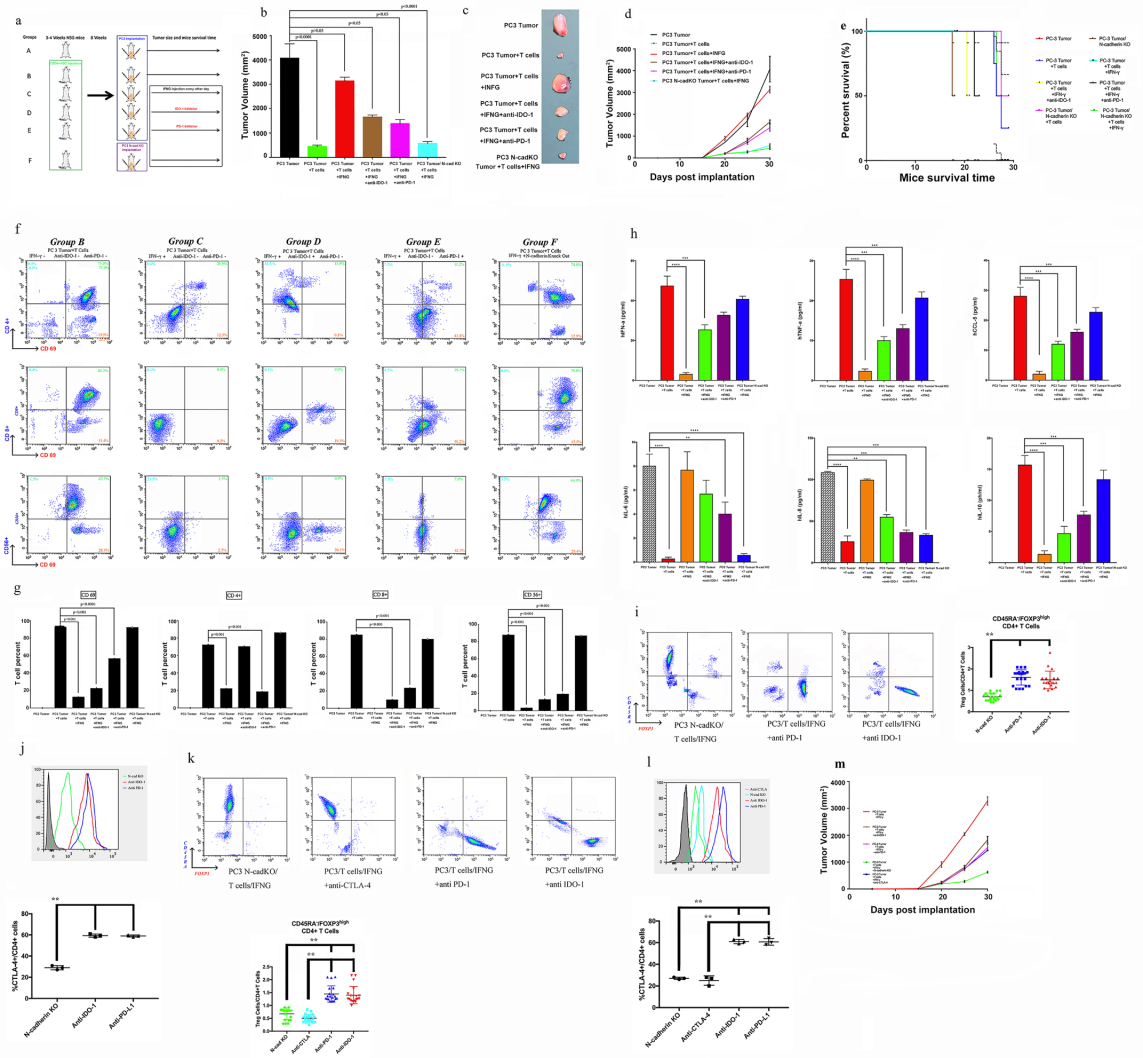
Evaluation of whether N-cadherin deletion reverses the IFN-g moderate immunosuppression induced by IFN-g. (A) Flow diagram of human immune cell engraftment and tumour implantation (groups A-E were implanted with PC3 vector control cells, only the last group contained PC3 N-cad- KO cells). We repeated the experiment twice (n=5 each time) and used CD34+ cells from two different donors. Final tumour volume (mm^3^) as measured percutaneously and corresponding images of dissected tumours (B, C). (D) Tumour growth curves through 30 days after implantation. (E) Survival time following tumour implantation (n=30);, and this experiment was repeated twice. (F, G) Tumour microenvironment TILs (human CD4+ cells, CD8+ T cells, CD56+ natural killer T cells and activation marker CD69) detected by flow cytometry. Single-cell suspensions were prepared from the tumours. We repeated the experiment twice (n=5 each time). (H) Sera were collected from NSG animals, and cytokines were assayed for the presence of human IFN-α, TNF-α, IL-8, IL-6, IL-10 and CCL5 using the SearchLight multiplex assay. (I) Representative contour plots of eTreg cells in mice mouse tumour samples classified according to the N-cadherin gene status and summaries are shown. TILs from tumour tissue samples were subjected to flow cytometry and identified as I, fraction I (naive Treg cells); II, fraction II (eTreg cells); and III, fraction III (non-Treg cells). (J) Representative histogram plots of CTLA-4 expression in eTreg cells in the N-cadherin-knockout group and anti-PD-1 and anti-IDO-1 groups and the summary are shown. TILs from tumour tissue samples were subjected to flow cytometry. (K) Representative contour plots of eTreg cells in mouse tumour samples in the N-cadherin-knockout group, anti-CTLA-4 group, and anti-PD-1 and anti-IDO-1 groups. (L) Representative histogram plots of CTLA-4 expression in eTreg cells in the N-cadherin-knockout group, anti-CTLA-4 group, and anti-PD-1 and anti-IDO-1 groups and the summary are shown. (M) The tumour growth curves of the indicated groups (with or without anti-CTLA-4) are shown. NC, negative control; bars, mean; error bars; *p < 0.05; and **p < 0.01.

**Figure 2:**
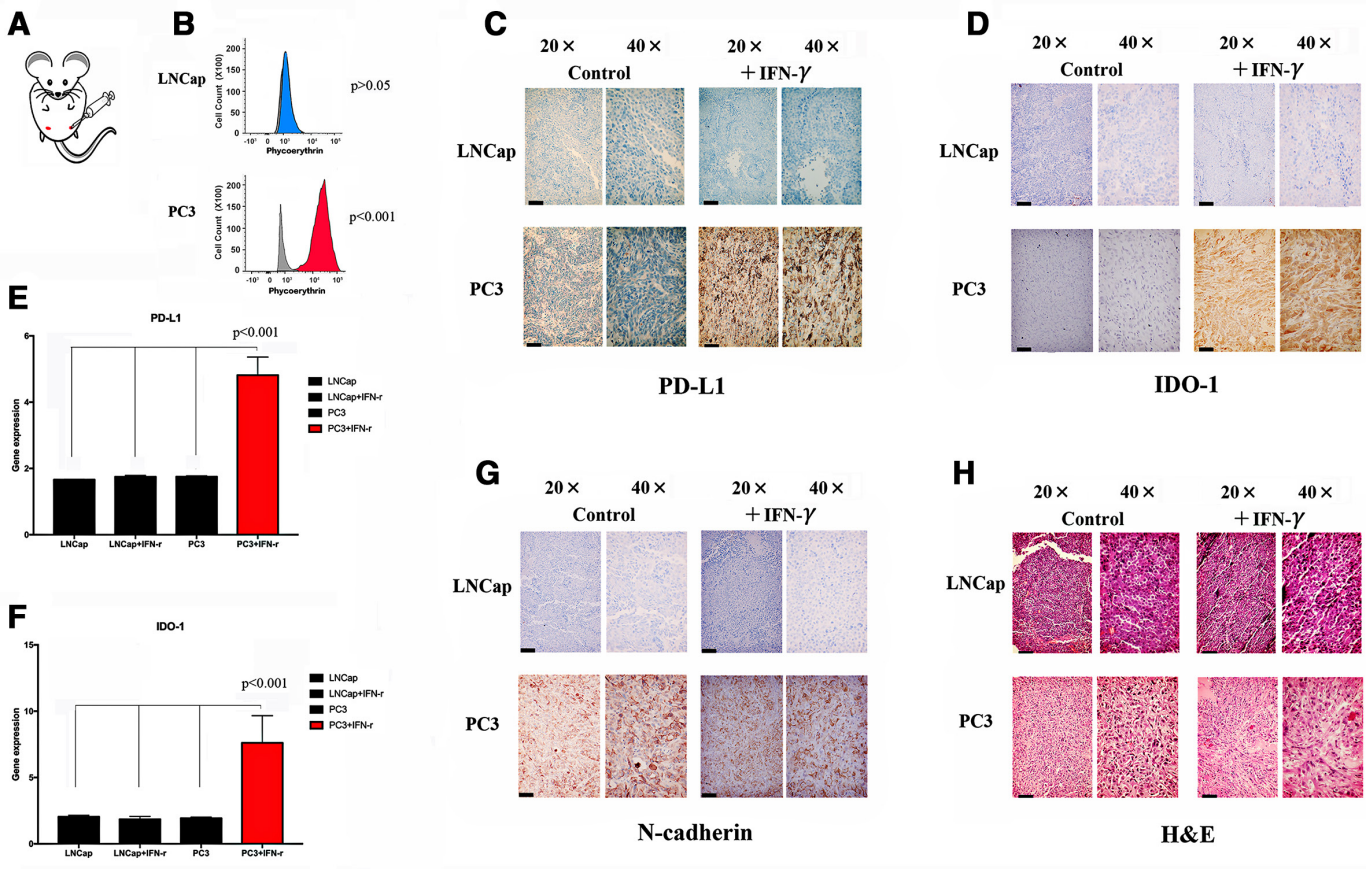
Expression of N-cad, IDO-1 and PD-L1 in mouse (n=5) tumors. (A) Tumor implantation site. (B) Flow cytometry analysis of mouse tumor cells after tissue dissociation. PD-L1 can be observed in PC3 cells under IFN-γ stimulation. (C) IHC staining for PD-L1 in different tumors; the black bars in the IHC image are 200 µm. (D) IHC staining for IDO-1 in different tumors; the black bars in the IHC image are 200 µm. (E) Expression of the PD-L1 mRNA in different tumors after tissue dissociation and RNA extraction. (F) Expression of the IDO-1 mRNA in different tumors. (G) IHC staining for IDO-1 in different tumors; the black bars in the IHC image are 200 µm. (H) H&E staining in mouse tissues; the black bars in the IHC image are 200 µm. IDO-1, indole amine 2,3-dioxygenase; IFN-γ, interferon gamma; IHC, immunohistochemistry; mRNA, messenger RNA; N-cad, N-cadherin; PD-L1, programmed death ligand-1.

We also performed an in vivo experiment to test the association between N-cadherin and immunosuppression and to better characterize the effect of N-cadherin on immunosuppression. We first implanted tumor cells in an inguinal site (figure 2A); PC3 cells were used in one group, while LNCap cells were used in the other group. Each group received IFN-γ as a treatment or phosphate-buffered saline (PBS) as a control after the tumors grew to 1 cm^3^. We collected the tumors at 48 hours after treatment administration (the animals were sacrificed by carbon dioxide (CO_2_) inhalation). Some tissues were embedded in paraffin, some were dissociated into single cells for flow cytometry analysis, and other tissues were used for RNA extraction for PCR. PD-L1 expression was examined using flow cytometry and was inducible only in the PC3 group (figure 2B). The evaluation of IHC staining revealed that only the PC3 tumor-bearing mice produced PD-L1/IDO-1 following IFN-γ stimulation (figure 2C, D). Similar results were obtained with PCR (figure 2E, F). Interestingly, as shown in figure 2G, N-cadherin expression was observed only in PC3 cells. Additionally, figure 2H shows H&E staining of mouse tissues.

Overall, the increased accumulation of N-cadherin was associated with higher expression of the checkpoint molecules PD-L1/IDO-1. We next aimed to evaluate whether the EMT and immunosuppression could mutually modulate each other.

### The mechanism by which N-cadherin and immunosuppression mutually regulate each other

**Figure 3:**
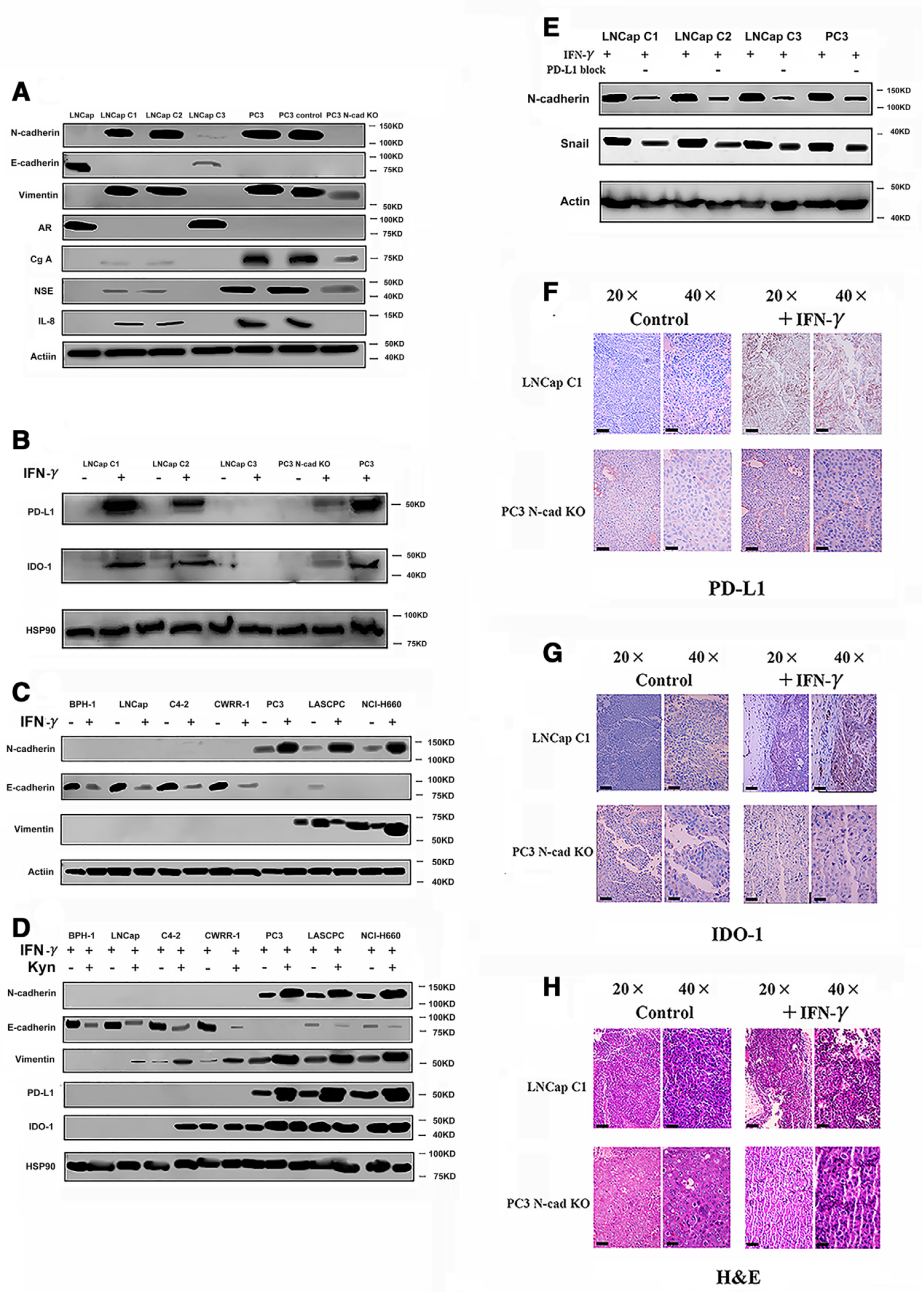
Evaluation of the effects of N-cad on the expression of PD-L1 and IDO-1 (five mice). (A) EMT and neuroendocrine (NE) phenotypes in N-cad-overexpressing cells and N-cad knockout (N-cad KO) cells. We used the LNCap, PC3 and PC3 vector (scrambled smallinterfering RNA) cells as the control. (B) PD-L1 and IDO-1 expression in N-cad-overexpressing cells and N-cad KO cells after treatment with IFN-γ. PC3 cells were used as a positive control. (C) Changes in the levels of N-cad in cell lines after treatment with IFN-γ. (D) Changes in the expression of PD-L1, IDO-1 and EMT markers after treatment with kynurenine. (E) Changes in the levels of EMT markers after treatment with the PD-L1 neutralizing antibody (“-” means PD-L1 block, blank means use PBS as blank control). (F) IHC staining for PD-L1 in different mice (n=5) tumors; the black bars in the IHC image are 200 µm. (G) IHC staining for IDO-1 in different mouse (n=5) tumors; the black bars in the IHC image are 200 µm. (H) H&E staining in mouse tissues; the black bars in the IHC image are 200 µm. AR, androgen receptor; Cg-A, Chromogranin-A; EMT, epithelial-mesenchymaltransition; IDO-1, indole amine 2,3-dioxygenase; IFN-γ, interferon gamma; IHC, immunohistochemistry; IL-8, interleukin-8; N-cad, N-cadherin; NSE, neuron-specific enolase; PBS, phosphate-bufferedsaline; PD-L1, programmed death ligand-1.

First, we compared the phenotypes of N-cadherin-overexpressing and N-cadherin knockout cells. The N-cadherin-expressing LNCap lines (LNCap C1, C2 and C3) were constructed by the team of Robert Reiter via the N-cadherin knock in.^19^ As shown in figure 3A and a previous study,^19^ LNCap C1 and C2 cells concomitantly lost E-cadherin and androgenreceptor expression and gained EMT and neuroendocrine differentiation phenotypes, while the LNCap subline (C3) retained E-cadherin expression and did not exhibit morphological changes (although we observed a low level of N-cadherin expression in LNCap C3 cells, E-cadherin was also expressed at higher levels than N-cadherin; therefore, we considered this cell line as a partial EMT cell line). Thus, only LNCap C1 and LNCap C2 cells produced PD-L1 and IDO-1 in response to IFN-γ (figure 3B and online supplemental figure 2A, B). In summary, N-cadherin altered the EMT process and then modulated IFN-γ signaling in cells. The LNCap C1 and C2 cells were characterized by a significant increase in growth compared with the LNCap C3 and LNCap parental cells (online supplemental figure 3A). Moreover, N-cadherin knockout decreased the growth of PC3 cells (online supplemental figure 3A). This finding further indicates that accelerated growth was mediated by N-cadherin. After N-cadherin was silenced, PD-L1 and IDO-1 expression almost completely disappeared, while neuroendocrine differentiation and EMT were reversed to some extent, and interleukin-8 (IL-8) expression was not detected (figure 3A). As shown in a previous study, IL-8 is a biomarker of N-cadherin signaling,^19^ and IL-8 deletion reversed EMT in the present study (online supplemental figure 3B), consistent with previous findings.^13 15 19^

10.1136/jitc-2020-002138.supp5 Supplementary data

10.1136/jitc-2020-002138.supp6 Supplementary data

We treated cell lines that represent benign prostate tissue and different prostate cancer types with IFN-γ and then evaluated the expression of the EMT markers N-cadherin, E-cadherin and vimentin to elucidate the underlying mechanism. N-cadherin and vimentin were upregulated in response to IFN-γ in the N-cadherin (N-cad)-activated cell lines (figure 3C), while E-cadherin expression was downregulated (figure 3C). However, IFN-γ did not induce N-cad-inactivated cells to express N-cadherin (figure 3C). The mechanism by which IFN-γ activates EMT remains unclear, and whether N-cadherin is able to regulate the PD-L1 and IDO-1 expression signature is unknown. Therefore, we evaluated whether IDO-1 and PD-L1 were able to affect the EMT status in these cells. IDO-1 is an enzyme involved in tryptamine metabolism that mediates the conversion of tryptamine into kynurenine. Hence, using western blotting, we detected whether the local accumulation of kynurenine enhanced the EMT process. Stimulation with 100 μmol/L kynurenine significantly increased the expression of N-cadherin and vimentin, while E-cadherin expression decreased (figure 3D). This result is consistent with the findings reported by Kolijn *et al*.^15^ Additionally, kynurenine and IFN-γ treatments failed to accelerate cell growth (online supplemental figure 3C), and thus the phenotypic change directly resulted from stimulation instead of rapid cell growth. Another interesting finding is that PD-L1 expression also increased after the kynurenine treatment, which may also explain why IDO-1 and PD-L1 expression were simultaneously increased in figure 1I; however, IDO-1 production did not significantly change (figure 3D), which differs from the results reported by Kolijn *et al*.^15^ Indeed, we tested the EMT status of cells treated with a PD-L1 neutralizing antibody by measuring N-cadherin and Snail expression. The activation of the EMT was reduced after treatment with the PD-L1 neutralizing antibody (figure 3E). Thus, IFN-γ not only induces immunosuppression but also promotes EMT. The mechanism by which immunosuppression alters EMT includes the expression of N-cadherin.

To support our proposal that N-cadherin should be considered a therapeutic target, we wished to acquire additional evidence from in vivo experiments, and thus, we constructed a new tumor-bearing mouse model. We implanted PC3 N-cad knockout (N-cad-KO) cells and LNCap C1 cells into NOD SCID gamma (NSG) mice. The tumor growth status was monitored for 4 to 5 weeks, in which the mice received two injections of IFN-γ, (the experimental group was intraperitoneally injected with 10 µg of IFN-γ in 200 mL of medium one time per day for two consecutive days; PBS was used in control group). Tumors were excised after IFN-γ treatment and processed for IHC staining to detect PD-L1 and IDO-1 expression. As shown in figure 3F–H, PD-L1 and IDO-1 expression was detectable in the LNCap C1 tumor sample after stimulation with IFN-γ, while PC3-derived tumors lacking N-cadherin no longer expressed PD-L1 and IDO-1. The anti-immunosuppressive effect of N-cadherin was identified by comparing those models. Indeed, the results presented in online supplemental figure 3D, E suggest rapid tumor growth in the N-cadherin-activated LNCap tumor-bearing mice, whereas the N-cad-KO PC3 model was characterized by a slight decrease in tumor growth. However, at the end of the observation period, the tumor sizes of PC3 (with or without N-cad-KO) were still significantly larger than those of the tumors (PC3 with or without N-cad-KO) treated with T cells (online supplemental figure 3E).

### The IFNGR/JAK/STAT pathway regulates the expression of PD-L1 and IDO-1

**Figure 4:**
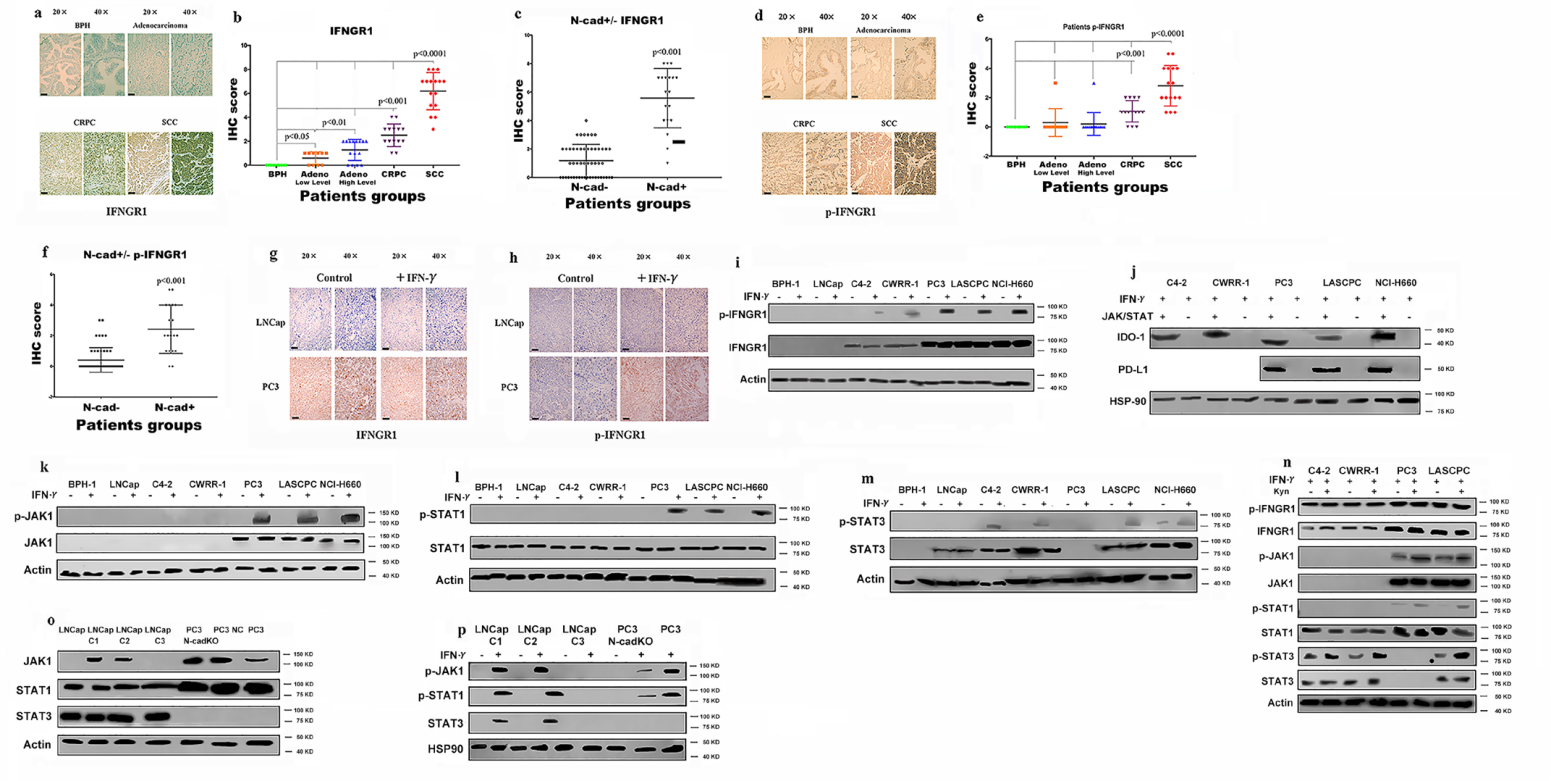
Exploration of the mechanism by which IFNGR1 regulates PD-L1 and IDO-1 expression. IHC samples including 30 benign samples, 15 low-level adenocarcinoma, 15 high-level adenocarcinoma, 18 CRPC samples and 16 SCC samples. (A) IHC staining for IFNGR1 in benign, adenocarcinoma, CRPC and SCC tissue samples; the black bars in the IHC image are 200 µm. (B) IHC scores for IFNGR1 expression in different tumors. (C) IFNGR1 expression in N-cad-positive and N-cad-negative tissues. (D) IHC staining for p-IFNGR1 in benign, adenocarcinoma, CRPC and SCC tissue samples; the black bars in the IHC image are 200 µm. (E) IHC scores for p-IFNGR1 levels in different tumors. (F) p-IFNGR1 levels in N-cad-positive and N-cad-negative cases. (G) IHC staining for IFNGR1 in mouse (n=5) tissue samples, the black bars in the IHC view are 200 µm. (H) IHC staining for p-IFNGR1 in mouse (n=5) tissue samples; the black bars in the IHC image are 200 µm. (I) Levels of IFNGR1/p-IFNGR1 in different cell lines. N-cadherin regulates PD-L1 and IDO-1 expression by modulating the JAK/STAT pathway. (J) Expression of PD-L1 and IDO-1 after blockade of the JAK/STAT pathway. (K, L, and M) Activation of the JAK/STAT pathway and PD-L1/IDO-1 expression. (N) Activation of the IFNGR/JAK/STAT pathway after treatment with Kyn. (O) The expression of JAK1/JAK3 in N-cad-overexpressing and N-cad-KO cells. (P) Activation of the IFNGR/JAK/STAT pathway in N-cad-overexpressing and N-cad KO cells treated with IFN-γ. PC3 cells were also used as a positive control. CRPC, castration-resistant prostate cancer; IDO-1, indole amine 2,3-dioxygenase; IFN-γ, interferon gamma; IHC, immunohistochemistry; Kyn, kynurenine; N-cad, N-cadherin; N-cad KO, N-cad knockout; PD-L1, programmed death ligand-1; SCC, small cell neuroendocrine prostate cancer.

We examined the mechanism of PD-L1 and IDO-1 expression, as well as the role of N-cadherin in this process. First, we focused on IFNGR1 because IFN-γ primarily regulates PD-L1 and IDO-1 expression through IFNGR1.^33^ The IHC results verified our hypothesis, as IFNGR1 was expressed at significantly higher levels in the SCC group, although some staining was also observed in the CRPC group (figure 4A, B). We also compared IFNGR1 expression in N-cadherin-positive cases and N-cadherin-negative cases, and the results showed increased expression of IFNGR1 in N-cadherin-positive tissues (figure 4C). Similar results were observed for p-IFNGR1, which is the activated form of IFNGR1, with the highest level detected in the SCC group (figure 4D, E). The level of p-IFNGR1 was also increased in the N-cadherin-positive tissues (figure 4F). Additionally, IHC staining of tissues from tumor-bearing mice revealed IFNGR1 expression only in the PC3 group, and IFNGR1 was activated only by IFN-γ in this group (figure 4G, H). As shown in figure 4I and online supplemental figure 4A, IFNGR1 was expressed at higher levels in N-cadherin-activated cell lines and was activated only by IFN-γ in the CRPC and SCC cell lines (N-cadherin-activated cell lines). All cell lines stimulated with IFN-γ produced IDO-1, but only N-cadherin-activated cell lines, which contain higher levels of p-IFNGR1, produced PD-L1 (figure 1F).

10.1136/jitc-2020-002138.supp7 Supplementary data

Moreover, as shown in figure 4J, PD-L1 and IDO-1 were no longer expressed when the entire JAK/STAT pathway was blocked. We confirmed that PD-L1 and IDO-1 expression was mediated by the JAK/STAT pathway, and therefore, we explored the effects of different proteins in the JAK/STAT pathway on the expression of PD-L1 and IDO-1. Generally, PD-L1 and IDO-1 are regulated by STAT1 or STAT3;^33^ thus, we analyzed the levels of the total and phosphorylated STAT1/3 proteins. JAK1 was expressed and activated only in SCC cell lines, and STAT1 was activated only in those cell lines (figure 4K and online supplemental figure 4A), although STAT1 was expressed in all cells (figure 4L and online supplemental figure 4A). In addition, when IDO-1 was expressed in CRPC cell lines, it was not regulated by STAT1 but rather STAT3, which was confirmed by the levels of STAT3 and p-STAT3. In CRPC cells, STAT3 was phosphorylated in response to IFN-γ (figure 4M), while STAT1 was not activated in response to IFN-γ (figure 4L). Indeed, STAT3 inhibition blocked the production of IDO-1 in CRPC, while it decreased only IDO-1 expression in SCC samples, and PD-L1 expression was not changed, as shown in online supplemental figure 4B. Thus, STAT3 does not regulate PD-L1 expression. Another interesting finding is that although STAT3 was not expressed in PC3 cells, PC3 cells were still able to produce IDO-1 and PD-L1 (figures 1F and 4M), indicating that the JAK1/STAT1 pathway regulated the expression of both PD-L1 and IDO-1. In addition, STAT3 was constitutively activated in the NCI-H660 cell line, which explained why IDO-1 was expressed in these cells without any treatment, while PD-L1 was not (figure 1F). Indeed, after blocking STAT1 signaling, IDO-1 expression decreased and PD-L1 expression was no longer detected in SCNE cell lines (online supplemental figure 4C).

We also evaluated the effect of kynurenine on the JAK/STAT pathway and found that kynurenine increased the phosphorylation of JAK1, STAT1 and STAT3, while the total protein levels did not change. Based on these results, IDO-1 increased the activation of the JAK/STAT pathway (figure 4N) and PD-L1 expression (figure 3E). These results and the data shown in figure 1I may explain the findings of other studies showing that PD-L1 and IDO-1 are always coexpressed in settings of immunosuppression.^34^ Then, we compared JAK1 expression between N-cadherin-overexpressing and N-cadherin-KO cell lines to explore why PD-L1/IDO-1 expression was induced in LNCap C1 and LNCap C2 cells. As shown in figure 4O, P and online supplemental figure 4D, JAK1 was expressed in LNCap C1 and LNCap C2 cells, but not in LNCap C3 cells, and N-cadherin-KO in PC3 cells decreased the levels of JAK1 and p-JAK1. Examination of the levels of phosphorylated proteins (figure 4P) revealed that the JAK/STAT pathway was activated in LNCap C1 and C2 cells stimulated with IFN-γ, supporting the data shown in figure 3E.

### N-cadherin deletion reverses the moderate immunosuppression induced by IFN-γ

According to the experimental paradigm, CD45+ immune cells were transferred to reconstitute the animal model (figure 5A). We divided the mice into five groups, groups A to E, in which we implanted the PC3 vector tumor cells as a control and used PC3 N-cad-KO cells in the last group (figure 5A). In agreement with several previous reports,^9 35 36^ the presence of human CD45+ cells in the blood was readily detectable by week 6 (mean, 13.5%±1.18%), reaching percentages of approximately 25% by week 8 (online supplemental figure 5A). The hCD45+ subpopulations of cells were evaluated. Online supplemental figure 5B shows the cell lineage distribution (expressed as a percentage of hCD45+) of hCD3+ T cells, hCD20+ B cells, hCD56+ NKTs, hCD33 myeloid cells and hCD68+ cells in the blood, bone marrow and spleen. Additionally, as presented in figure 5B-D, the tumors from NSG mice receiving TIL treatment in group B (PC3 tumor+T cells) were significantly smaller than those in the blank control group. In contrast, IFN-γ promoted rapid and aggressive tumor growth in group C (PC3 tumor+T cells+IFN-γ) compared with group B. To further characterize the immunotherapy, an IDO-1 inhibitor and PD-1 blocking antibody were applied. Tumor growth was attenuated in group D (PC3 tumor+T cells+IFN-γ+anti-IDO-1) and group E (PC3 tumor+T cells+IFN-γ+anti-PD-1) compared with group C (PC3 tumor+T cells+IFN-γ). Indeed, further observation showed that N-cadherin deletion (group F (PC3 N-cad-KO+T cells+IFN-γ)) was more effective than the IDO-1 inhibitor or PD-1 blocking antibody in reducing the tumor growth. As shown in figure 5E, N-cadherin deletion dramatically extended the survival time compared with mice receiving the IDO-1 inhibitor or PD-1 blocking antibody alone. The mechanism responsible for this phenomenon might be explained by the data shown in online supplemental figure 6, in which after N-cadherin deletion, both PD-L1 and IDO-1 were no longer induced by IFN-γ. N-cadherin can reduce the expression and activation of JAK-1, which remained active when PD-L1 or IDO-1 alone were inhibited (online supplemental figure 6).

10.1136/jitc-2020-002138.supp8 Supplementary data

10.1136/jitc-2020-002138.supp9 Supplementary data

To study the human cytokine and chemokine profiles in huPBL-NSG animals’ alteration after N-cadherin deletion, serum lymphocytes and TIL samples are collected for analysis. The presence of human T cells in the tumor were readily detectable (figure 5F, G). CD34+ hematopoietic stem cells (HSCs) can be differentiated into several subgroups (CD8+ T cells, CD4+ T cells and CD56+ natural killer T (NKT) cells) after interaction with human tumors. Human lymphocytes reduced the tumor size (figure 5A-C, F, G); however, PD-L1/IDO-1 attacked TILs and attenuated this process under IFN-γ pathway activation in group C (PC3 tumor+T cells+IFN-γ; figure 5A-C, F, G). IDO-1 blockade alone rescued only CD4+ T cells (figure 5F, G group D (PC3 tumor+T cells+IFN-γ+anti-IDO-1)), suggesting that PD-L1 could still damage CD8+ T and NKT cells. PD-L1 inhibition recovered only the expression of CD69 (figure 5F, G group E (PC3 tumor+T cells+IFN-γ+anti-PD-1)), which is the primary activation marker of T cells and can prevent the harmful effects of tryptamine deletion caused by IDO-1. Importantly, N-cadherin deletion rescued all T cell subgroups through blocking the IFN-γ pathway and improved TIL-related therapy (figure 5A-C, F, G, online supplemental figure 6, group F (PC3 N-cad-KO+T cells+IFN-γ)). The serum cytokine data are in agreement with the report by Roth *et al*,^10^ in which IL-6 and IL-8 decreased when TILs caused tumor shrinkage (figure 5H), and other cytokines (interferonalpha (IFN-α), TNF-α (tumornecrosis factor alpha), CCL5 and IL-10) were positively related to the T cells (figure 5H). Then, to quantitatively analyze Treg cells in the mouse model, TILs were subjected to flow cytometry and assessed based on the transient upregulation of FOXP3 expression on T cell receptor (TCR) stimulation of naive T cells.^37^ FOXP3+CD4+cells were divided into the following three subgroups according to the expression levels of the naive cell markers CD45RA and FOXP3:^38 39^ naive Treg cells (Fraction (Fr.) I: nTreg cells, CD45RA+FOXP3lowCD4+) with weak immunosuppressive function, effector Treg cells (Fr. II: eTreg cells, CD45RA–FOXP3highCD4+) with strong immunosuppressive function, and non-Treg cells (Fr. III: CD45RA–FOXP3lowCD4+) without immunosuppressive function. The frequency of eTreg cells and the expression of CTLA-4 in eTreg cells decreased in the N-cadherin-knockout group, while no changes were observed in the anti-PD-1 and anti-IDO-1 groups (figure 5, J). The frequency of eTreg cells decreased with the use of anti-CTLA-4 (figure 5K), along with a decrease in the expression of CTLA-4 in eTreg cells and a reduced tumor growth (figure 5L, M). Overall, knocking out N-cadherin was better in reducing the generation of eTreg cells than anti-PD-1 and anti-IDO-1. Next, we aimed to further job to explore the mechanism of how N-cadherin regulates the status of eTreg cells.

### N-cadherin knockout eliminates the metabolic advantage of Treg cells

**Figure 6:**
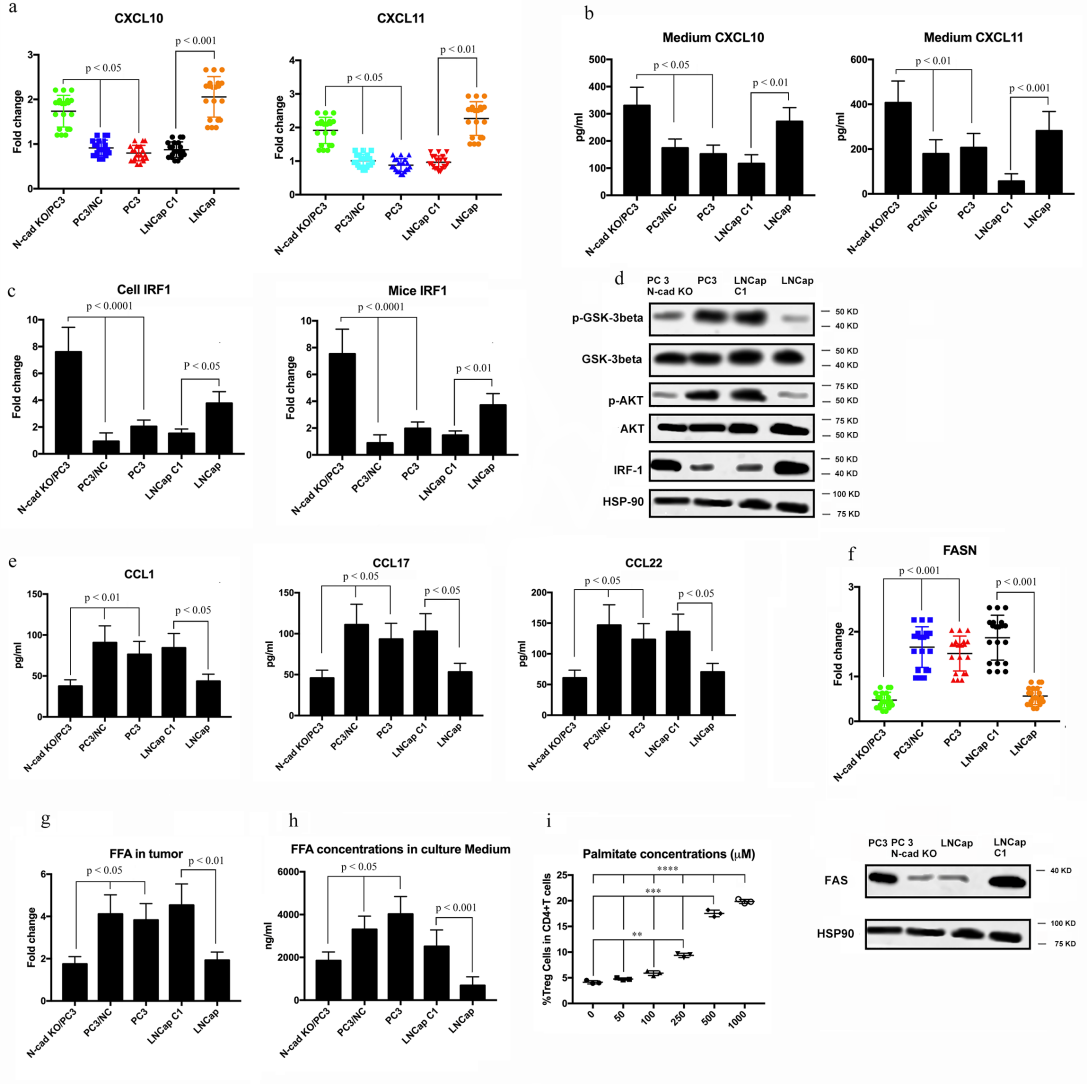
Evaluation of whether N-cadherin knockout impacts the production of Treg cells. (A) CXCL10 and CXCL11 expression levels in cell lines were examined by real-time qRT-PCR. Fold changes are shown. (B) The concentrations of CXCL10 and CXCL11 in the culture of cell lines were analysed by ELISA. Cells were cultured with RPMI medium containing 10% FBS. Then, 48 h later, the concentrations of CXCL10 and CXCL11 were examined. (C) IRF1 expression in cell lines and mouse tumour cells was evaluated by real-time qRT-PCR. Fold changes are shown. (D) The protein expression of members of the AKT-mTOR signalling pathways in cell lines was examined by western blotting. (E) The concentrations of CCL1, CCL17, and CCL22 in the culture medium were analysed by ELISA. Cells were cultured with RPMI medium containing 10% FBS. Then, 48 h later, the concentrations of CCL1, CCL17, and CCL22 were examined. (F) Fatty acid metabolism-related genes were compared using RNA-sequencing data. (G) FASN/FAS was examined by real-time qRT-PCR and western blotting. FFA concentrations in tumour samples (H) were evaluated using the Free Fatty Acid Quantification Kit and in culture medium (I) using LC-MS. Cell lines were cultured with FBS-free RPMI medium containing 10% lipids. Then, 48 h later, the concentrations of FFAs were examined.

The expression of CXCL10, CXCL11 and IRF1 was upregulated by N-cadherin-knockout (figure 6A–C). Overall, blocking N-cadherin increased the levels of effector T cell-recruiting chemokines such as CXCL10 and CXCL11 through an increase in IRF1 expression. In line with this fingding, the reduced phosphorylation of GSK-β induced by N-cad-KO synchronized appearance with the increase in IRF1 expression (figure 6D); this result is in agreement with the findings of Kumagai *et al*.^40^ Additionally, the protein expression of CCL1, CCL17, and CCL22, which reportedly recruit eTreg cells,^21 41^ also decreased after knocking out N-cadherin (figure 6E). To further elucidate the mechanisms by which N-cad-KO regulated eTreg cells, we performed Gene Set Enrichment Analysis of the RNA-sequencing data, which revealed that the gene set related to fatty acid metabolism was blocked in N-cad-KO PC3 cells (figure 6F). Consistent with the above result, the expression of FAS and FASN (the gene encoding FAS), which plays an important role in fatty acid synthesis,^42^ was lower in N-cad-KO PC3 cells (figure 6G), and tumors contained smaller amounts of free fatty acids (FFAs) after N-cad-KO in vivo (figure 6H). Accordingly, the total concentration of FFAs in the culture medium was lower in the N-cad-KO PC3 cells (figure 6I). The higher FFA production by N-cadherin-expressing cancer cells prompted us to explore whether the abundant FFAs contributed to the prolonged survival of Treg cells. To this end, we used low-glucose medium supplemented with increasing concentrations of palmitate to culture peripheral blood mononuclear cells from healthy individuals. The frequency of eTreg cells increased in a concentration-dependent manner (figure 6I). The results indicated that the AKT-mTOR signaling pathway may participate in the process of N-cadherin-induced increase in FFA production (figure 6D), which is in agreement with the research of Kumagai *et al*.^40^ Because we confirmed that IL-8 was regulated by N-cadherin, and our previous research showed that IL-8 can activate AKT/GSK-3β,^43^ we hypothesized that N-cad-KO can downregulate IL-8 expression and then block the AKT-mTOR pathway. First, we determined whether IL-8 knockout can decrease the expression of JAK-1, and the data shown in online supplemental figure 7A indicated that JAK-1 expression did not change after the IL-8 knockout. However, knocking out IL-8 decreased eTreg cells (online supplemental figure 7B). The expression of CXCL10, CXCL11 and IRF1 was upregulated by IL-8 knockout (online supplemental figure 7B-E), while the protein expression of CCL1, CCL17, and CCL22, which reportedly recruit eTreg cells, decreased after blocking IL-8 (online supplemental figure 7F), in agreement with the data in figure 6E. Additionally, the expression of FAS and FASN was decreased in IL-8-knockout PC3 cells (online supplemental figure 7G), and the total concentration of FFAs in the culture medium was lower in these cells (online supplemental figure 7H).

10.1136/jitc-2020-002138.supp10 Supplementary data

### ADH-1 plays the role of an adjuvant in TIL-related therapy in hNSG PC3-bearing animals

**Figure 7:**
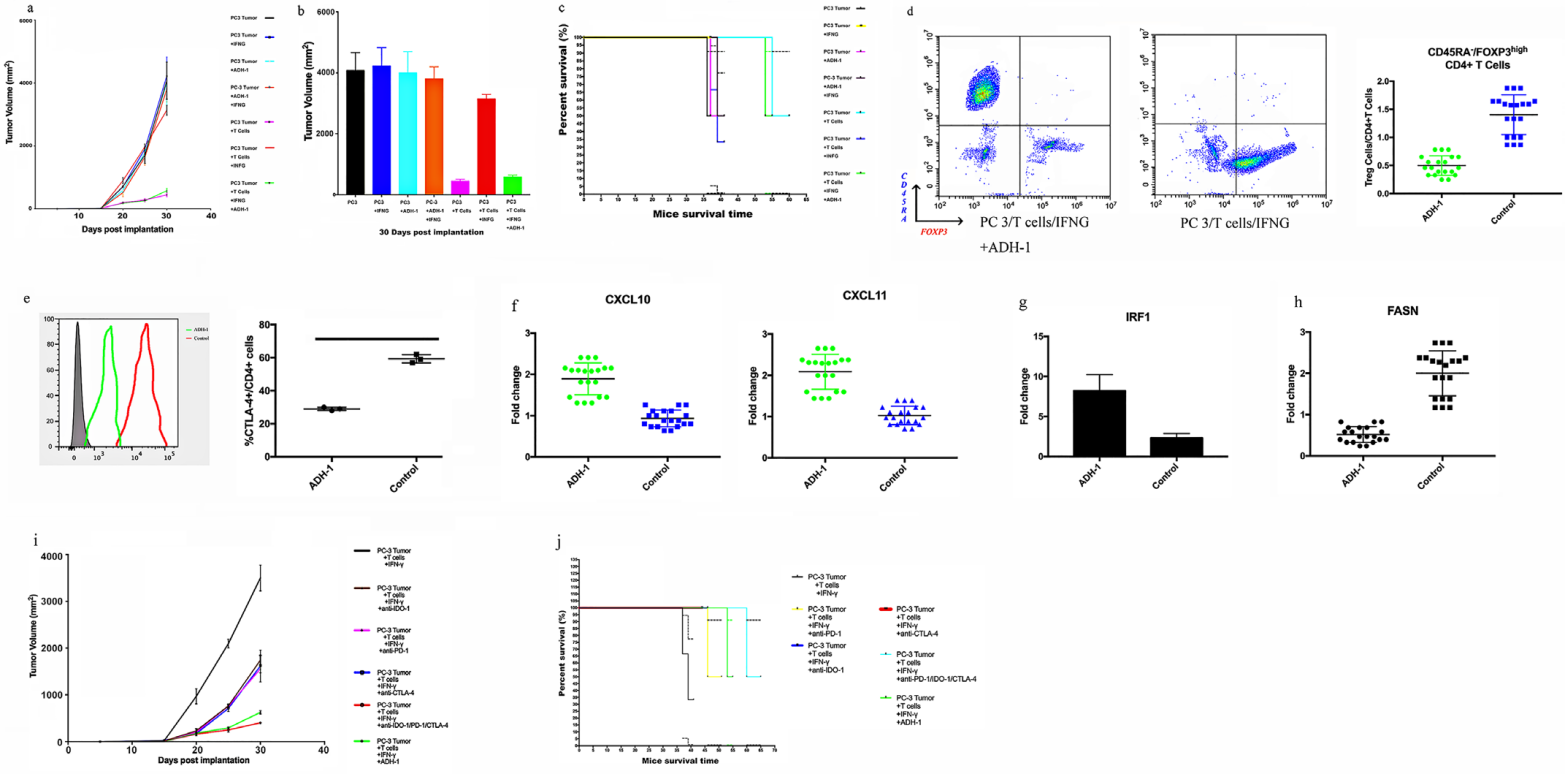
Evaluation of whether the ADH-1 can improve the effiencyefficacy of TIL-related therapy in hNSG PC3-bearing animals. (A) Preclinic treatment experiment with N-Ac-CHAVC-NH2 (designated ADH-1). Tumour growth curves through 30 days after implantation. (B) Final tumour volume (mm^3^) measured percutaneously and corresponding images of dissected tumours. (C) Survival time following tumour implantation (n=30);. tThis experiment was repeated twice. (D) Representative contour plots of eTreg cells in mouse tumour samples classified after using ADH-1 are shown. TILs from tumour tissue samples were subjected to flow cytometry and characterized as follows: I, fraction I (naive Treg cells); II, fraction II (eTreg cells); and III, fraction III (non-Treg cells). (E) Representative histogram plots of CTLA-4 expression in eTreg cells after using ADH-1 are shown. TILs from tumour tissue samples were subjected to flow cytometry. (F) CXCL10 and CXCL11 expression levels in mouse tumour cells were examined by real-time qRT-PCR. Fold changes are shown. (G) IRF1 expression in mouse tumour cells was evaluated with real-time qRT-PCR. Fold changes are shown. (H) FASN was examined with real-time qRT-PCR. (I) Tumour growth curves through 30 days after implantation. (J) Survival time following tumour implantation (n=30). This experiment was repeated twice.

Additionally, as presented in figure 7A, B, the tumors from the NSG mice receiving TIL treatment were significantly reduced in size compared with those in the blank control group. In contrast, IFN-γ promoted rapid and aggressive tumor growth, as shown in figure 5, indicating that IFN-γ can induce the expression of PD-L1 and IDO-1 and suppress TILs. When we used ADH-1 and TILs as combination therapy, IFN-γ-mediated immunosuppression was no longer be able to obstruct the efficiency of the TIL-related treatment. We also established additional groups as a control: tumor treatment with IFN-γ alone; ADH-1 alone; or IFN-γ and ADH-1 together. No impact on tumor growth was observed, in agreement with the study by Li *et al*,^44^ which excluded the impact of these drugs on tumors and allowed us to focus on the immune effect. The results presented in figure 7C show that ADH-1 significantly extended the survival time with the assistance of TILs avoiding damage by the IFN-γ pathway. The frequency of eTreg cells and the expression of CTLA-4 in eTreg cells were decreased in the ADH-1 group (figure 7D, E), in agreement with the data in figure 5. The expression of CXCL10, CXCL11 and IRF1 was upregulated after using ADH-1 (figure 7F, G), which was consistent with the data shown in figure 6A–C. Indeed, the expression levels of FASN were lower in the ADH-1 group (figure 7H), in agreement with the data in figure 6G. More importantly, the efficiency of ADH-1 in decreasing the tumor size and prolonging mouse survival time was better than those of anti-PD-1, anti-IDO-1 and anti-CTLA-4 when used individually or in combination (figure 7I, J).

## Discussion

In recent years, several studies have demonstrated the successful establishment of TIL cultures from multiple tumor types, including melanoma, lung, breast, pancreatic, renal and bladder cancer^45–48^ but not prostate cancer, while previous works have described the immunosuppressive microenvironment of prostate cancer.^49 50^ The discovery of a new method to protect lymphocytes from immune invasion in the microenvironment of prostate cancer is urgently needed.

The N-cadherin antagonist ADH-1 has been shown to be an anti-angiogenic agent, and several clinic trials have revealed that it can be used as an antitumor drug.^23–25^ According to a recent paper, N-cadherin regulates the EMT process in advanced cancers.^19^ Additionally, immunosuppression is linked to EMT.^27–31^ As previously reported, EMT enforces immunosuppression and helps cancer cells escape immunosurveillance, which is known to play a major role in tumor invasion.^14–18^ Therefore, we propose that N-cadherin mediates immunosuppression through the initiation of the EMT. First, we tested patient samples; some patients in the SCC group exhibited high levels of PD-L1/IDO-1, while some patients exhibited low levels, and some showed a lack of expression. This finding is in agreement with some reports.^15 51 52^ Then, we systematically profiled in vitro and in vivo models and performed a preclinical study to reveal a potential new targeted treatment strategy involving N-cadherin and the molecular mechanism by which N-cadherin regulates immunosuppression. The core finding of this research is that N-cadherin deletion can improve TIL-related treatment by attenuating PD-L1, IDO-1 and eTreg cell generation. When we used N-cadherin antagonists ADH-1 with TIL-related treatment as combination therapy, the tumor size was reduced, and the survival period of mice was extended. We believe these results provide some experimental evidence for future clinical use.

TILs are present in all tumors and represent the endogenous immune response to cancer growth.^6–8^ Tumor cells in the human body are usually eliminated by natural immune attack of TILs, but cancer cells can escape this process via various mechanisms. For instance, PD-L1 combined with PD-1 in T cells induces apoptosis, and IDO-1 promotes the consumption of tryptamine. Some researchers have proposed that PD-1 blockade induces an incomplete rescue in cancers overexpressing this protein. Furthermore, other studies^6^ have documented synergistic effects of IDO-1 and PD-L1 on tumor immune evasion, which is consistent with our data. Thus, a combination treatment targeting both IDO-1 and PD-L1 should be explored; however, the best method would be to block the upstream pathway that regulates the expression of immunosuppression-related factors. Therefore, the first step in our study was to validate the role of N-cadherin in the secretion of PD-L1/IDO-1. The highest levels of N-cadherin and PD-L1/IDO-1 were observed in SCPC, and PD-L1/IDO-1 were expressed at higher levels in N-cadherin-positive tumors. Recently, a new molecular link between EMT-mediated upregulation of PD-L1 expression and CD8+ TIL immunosuppression has been established in human lung cancer.^53^ EMT directly regulates the expression of PD-L1 and is associated with several other checkpoint ligands^53 54^; however, our study is the first to show that PD-L1 expression is increased by the EMT marker N-cadherin in prostate cancer. Kolijn *et al*^15^ also proposed that IDO-1 regulates N-cadherin expression, which may prompt prostate cancer cells to produce IDO-1, but the authors did not evaluate PD-L1 and provided only mRNA and not protein data. We extended this finding, revealing the mutual regulation between EMT and immune checkpoint molecules and verifying this finding in patient-derived tissues, cells and a tumor-bearing mouse model. We also observed coordinated expression of IDO-1 and PD-L1. Thus, the N-cadherin block is an optimal therapy because it disrupts PD-L1 and IDO-1 expression and abrogates the immunosuppressive effect elicited by IFN-γ pathways that are active in the tumor microenvironment. This point has been demonstrated by a preclinical study in humanized NSG animals, which provides a unique opportunity to study immunotherapy in vivo.^9 10^ To achieve this goal, the construction of humanized models by injecting mice with human CD34+ HSCs can better simulate the human immune system and are currently commercially available,^9^ which is why we chose this protocol instead of donor PBL injection.^10^ Our data showed that N-cadherin deletion attenuated the immunosuppression induced by the PD-L1/IDO-1 pathway in vivo. Accordingly, Kumagai *et al* demonstrated that abundant FFA production by the PI3K-AKT-mTOR signaling pathways provides a metabolic advantage for the survival and immunosuppressive function of Treg cells.^40^ In our study, blocking N-cadherin or downregulating IL-8 attenuated the metabolic advantage and the immunosuppression caused by eTreg cells. However, there are some limitations to acknowledge: (1) how N-cadherin regulates FFA production is unknown and requires further research in prostate cancer; (2) if Tregs have reduced proliferation or survival, differential homing, or distinct differentiation, among other possibilities, still requires further research in the future; and (3) the impact of N-cadherin on Treg also been reported in other cancers^55^ and may need further research in prostate cancer.

To extend our research, we used the N-cadherin antagonist ADH-1 as an adjuvant therapy to improve the efficiency of TIL-related treatment. This combination therapy may provide some new insights into research regarding TIL-related treatment in prostate cancer.^56^

Furthermore, we also revealed how N-cadherin modulates the IFNGR-JAK1-STAT1 pathway, which decreases antitumor immunity by regulating PD-L1/IDO-1 secretion. N-cadherin increased JAK1 expression. Moreover, activation of the JAK1/STAT1 pathway was associated with increased expression of both PD-L1 and IDO-1. In contrast, JAK2-STAT3 signaling was linked to only IDO-1 expression. Genomic loss of JAK1 occurs in some adenocarcinoma and CRPC cell lines,^57^ explaining why some cell lines, particularly adenocarcinoma cell lines, with a deficient IFN-γ response fail to produce PD-L1/IDO-1. The expression of JAK1/2, STAT3 and PD-L1 increases during EMT, which has been reported in lung cancer^58^ and is consistent with our findings that JAK1 expression was rescued in LNCap C1/C2 cell lines expressing JAK1. Our data explain the active N-cadherin feedback loop between immunosuppression and EMT. These findings provide insights into the molecules and signaling pathways involved in the interaction between EMT and other immune processes, which will hopefully promote the development of different therapeutic strategies aimed at enhancing or suppressing specific EMT functions, depending on the pathological context. Overall, we defined a positive feedback loop between EMT and immune checkpoint protein expression that is initiated by N-cadherin. Moreover, strategies targeting N-cadherin significantly reverse immunosuppression, which is a very innovative discovery. The N-cadherin inhibitor ADH-1 did not show antitumor potential in a PC3 xenograft tumor model in the research by Li *et al*.^44^ However, ADH-1 could reduce the immunosuppression mediated by IFN-γ according to our data, and the team of Robert Reiter^19^ reported that N-cadherin-targeted antibodies delayed CRPC progression and growth. Indeed, our results also suggest that the mechanism of the metabolic advantage mediated by the N-cadherin-IL-8-AKT-mTOR pathway observed in our study may provide a helpful explanation for the development of immunosuppressive therapy in prostate cancer. All of these results raise the possibility that N-cadherin-blocking therapy may be translatable to the clinic.

Although we used multiple model systems and human tissues, our study still had some limitations. For example, IDO-2 and TDO2 are also involved in tryptamine degradation, but the roles of these two enzymes in EMT remain to be elucidated. The PC3 cell line is also controversial. PC3 is a unique cell line, and we usually do not classify it as SCC, although it possesses some neuroendocrine phenotypic characteristics, such as the expression of neuron-specific enolase. However, we sought to explore the association between N-cadherin and immunosuppression, and thus we considered PC3 cells as an N-cad-positive cell line that expresses some neuroendocrine markers. We also used two verified NE cell lines, LASCPC-01 and NCI-H660, in this experiment to provide additional evidence.

## Conclusions

In summary, N-cadherin rescues the expression of JAK1 and promotes the IFN-γ-induced production of PD-L1 and IDO-1. Indeed, N-cadherin deletion leads to the decline of PD-L1/IDO-1 expression and protects TILs from the damage caused by those immunosuppression factors. Preclinical research has revealed that N-cadherin antagonists ADH-1 can improve the efficiency of TIL-related treatment.

## Materials and methods

### Patients and tissue samples

Several (TMAs) were constructed. TMAs containing both non-tumor and tumor tissues were constructed with prostatectomy specimens obtained from 30 patients with localized prostate adenocarcinoma. Three cores were obtained from the non-tumor and tumor areas of each prostatectomy specimen and were incorporated into the TMA. A CRPC TMA was constructed using CRPC tissue samples from 18 patients who were histologically diagnosed with adenocarcinoma but failed to respond to hormone therapy. These tissue samples were derived from tissue collected during transurethral resection for the relief of obstructive symptoms and were incorporated into the CRPC TMA. An SCC TMA was constructed from 16 primary SCC samples collected at Duke University Hospital. All samples were collected from patients who provided informed consent, and all related procedures were performed with the approval of the internal review and ethics boards of the indicated hospitals.

### Immunohistochemistry of tissue samples

In preparation for IHC, all sections (including the slides containing target samples and positive and negative control slides) were deparaffinized, rehydrated and boiled in a water bath for 40 min in citrate buffer (pH 6.0) before antibody staining. Then, the slides were incubated with primary antibodies (the optimized dilution was previously determined) for 1 hour at room temperature. Horseradish peroxidase-conjugated secondary antibodies (Dako, EnVision Kit) were applied for 30 min and visualized with diaminobenzidine after an incubation for 30 min incubation at room temperature.

### Cell culture and cell treatment

The benign prostate cell line BPH-1 and the prostate cancer cell lines LNCap, C4-2, CWRR-1, PC3 and NC-IH660 were obtained from ATCC. The LASCPC-01 cell line was obtained from Dr Owen N Witte.^59^ The NC-IH660 and LASCPC-01 cells were cultured in HITES medium, while the LNCap, C4-2, CWRR-1 and PC3 cells were cultured in RPMI medium supplemented with 10% fetal bovine serum (FBS) and 1% penicillin. BPH-1 cells were cultured in RPMI medium supplemented with 20% FBS and 1% penicillin. The LNCap C1/C2/C3/LC1 cell lines and the single cloned N-cadherin-knockout PC3 cell line (PC3 N-cad-KO) was obtained from Dr Robert E Reiter (shorthairpin RNA (shRNA) against N-cadherin was subcloned into the GFP-positive lentiviral vector FG-12 (Addgene) to generate FG12-shNcad, while scrambled shRNA was also used to generate the control vector). These cell lines were also cultured in RPMI medium supplemented with 10% FBS and 1% penicillin. All cell lines were maintained at 37°C in an atmosphere containing 5% CO_2_. When we tested the effects of IFN-γ, a JAK/STAT inhibitor, and kynurenine on the cells, we treated them with 50 mg/mL IFN-γ (PeproTech 300–02; Rocky Hill, New Jersey, USA) for 24 hours, 250 nM JAK/STAT inhibitor (Santa Cruz CAS 457081-03-7) or 100 μmol/L kynurenine in the medium for 48 hours. We also treated the cells with a PD-L1 neutralizing antibody (10 µg/mL) (Invitrogen, 16–5982–82) for 24 hours to inhibit PD-L1 expression.^58^ IL-8 inhibition was achieved as previously described.^43^ Briefly, cells were transfected with 100 nM DNA single clone plasmids containing a shRNA to silence IL-8 (category number C01001; GenePharma, Shanghai, China) using the transfection reagent Lipofectamine 3000 (Invitrogen, Carlsbad, California, USA) according to the manufacturer’s instructions.

### Western blot and ELISA analyses

After culturing, the cells were lysed, and the proteins were extracted using RIPA and pooled according to the manufacturer’s instructions. The extracted proteins were separated by 10% sodium dodecyl sulphate-polyacrylamide gel electrophoresis. The reactive protein bands were detected using the chemiluminescence method (ECL Plus western blot Detection System; Amersham Biosciences, Foster City, California, USA). The concentrations of human CXCL10, CXCL11, CCL1, CCL17, and CCL22 and murine CXCL10 and CXCL11 were examined with specific sandwich ELISAs according to the manufacturer’s instructions (R&D Systems, Minneapolis, Minnesota, USA).

## Real Time PCR

Total RNA was extracted using TRIzol reagent, and cDNA was synthesized using an iScriptTM cDNA Synthesis Kit (Bio-Rad, Richmond, California, USA). The housekeeping gene GAPDH was used as an internal control. The PCR cycles were performed using SYBR Premix Ex Taq II (Takara, Dalian, Liaoning, China) according to the manufacturer’s instructions. PCR was performed using a Bio-Rad iQ5 thermal cycler (Hercules, California, USA), and the quality of the resulting PCR products was monitored through post-PCR melting curve analysis.

### Experiments with tumor-bearing mice

Immunocompromised NSG (NOD.Cg-Prkdc Il2rg/SzJ) and nude (nu/nu) mice were obtained from The Jackson Laboratory. A total of 2×10^6^ LNCap/LNCap C1 or 1×10^6^ PC3/PC3 N-cad-KO cells were suspended in 0.1 mL of 1× RPMI 1640 medium supplemented with 10% FBS and 50% Matrigel (Corning) and then inoculated subcutaneously into the bilateral flanks of 3-week-old to 6-week-old male NSG mice. After the LNCap and PC3 xenografts grew to 1 cm^3^ (the tumor volume was calculated using the formula volume (mm^3^)=length×height^2^/2), the mice were divided into two groups. Each mouse in the first group was intraperitoneally injected with 10 µg of IFN-γ in 200 mL of Iscove’s Modified Dulbecco’s Medium (IMDM, Life Technologies, Grand Island, New York) one time per day for two consecutive days. Each mouse in the second group received PBS as a control. Forty-eight hour after IFN-γ or PBS injection, the mice were sacrificed, and the xenografts were harvested, after which they were fixed with 10% formalin and embedded in paraffin for subsequent analysis. Single cells were isolated from the xenografts; the tissue samples were completely minced and incubated overnight with an enzyme mixture for digestion (80 mg of collagenase type II in 40 mL of advanced DMEM with a 1/1000 dilution of a ROCK inhibitor and 1/1000 dilution of 1.0 M DHT). The cells were washed with fresh advanced DMEM prior to culture in 5 mL of TrpLE +5 µL of ROCK inhibitor in a 37°C incubator for 5 min. The cells were repeatedly pipetted up and down and cultured again to efficiently separate the clumps. The cells were then centrifuged and stored in PBS until the flow cytometry analysis or RNA extraction.

### Immune reconstitution in tumor-bearing mice

NSG mice aged 3 to 4 weeks were implanted with 3×10^4^ initial human CD34+ HSC (Stem Cell Technologies, Vancouver, British Columbia, Canada) via tail vein injection.^9^ PC3 vector (group A to E) or N-cad-KO (group F) prostate cancer cells (2×10^6^) were implanted into the bilateral flank by subcutaneous injection 8 weeks after HSC engraftment.^10^ Group A was implanted with tumor cells only and served as the blank control. Group B was implanted with HSCs without any other therapeutic strategy. The remaining groups were treated with IFN-γ (10 µg of IFN-γ in 200 mL of IMDM) every other day after tumor implantation. Group D was treated with an IDO-1 inhibitor (0.2% 1-methyltryptamine in the drinking water), and group E was treated with an anti-PD-1 antibody (10 mg/kg nivoluzumab) following a weekly schedule. Only group F was implanted with N-cad-KO PC3 cells. Each group included five mice. The peripheral blood of recipient mice was collected from the retro-orbital sinus and analyzed by flow cytometry. The survival time was recorded, and the tumor size was measured through the first 30 days after implantation. To further study the efficiency of N-cadherin-targeted therapy, we injected mice with ADH-1. The therapy group was treated with TILs, IFN-γ and intraperitoneal bolus injections of ADH-1 (200 mg/kg) 5 days a week^44^ and control groups were also included (PC3 tumor cells, PC3 tumor cells with ADH-1, PC3 tumor cells with IFN-γ, PC3 tumor cells with IFN-γ and ADH-1, PC3 tumor cells with TILs, and PC3 tumor cells with TILs and IFN-γ). The anti-CTLA-4 mAb (9H10) was obtained from BioLegend (San Diego, California, USA) and used as according to the protocol. After the mice were euthanized, tumors were collected for IHC or isolated into single cells as previously described. After single cell suspensions were acquired from the tumors, the expression of human TIL markers (CD4, CD8, CD56, CD69, CD45RA, FOXP3 and CTLA-4) was assessed by flow cytometry (fluorochrome-conjugated mAbs were obtained from BD Biosciences). Human cytokines (IFN-α, TNF-α, IL8, IL6, CCL5 and IL10) in the mouse serum samples were assayed by ELISA (Human cytokine ELISA kit, Biocompare, Newburyport, Massachusetts, USA). The human cytokine array was quantified using a commercial SearchLight multiplex assay (Aushon Biosystems Inc, Billerica, Massachusetts, USA).

### Quantification and statistical analysis

All data are presented as the mean±SD and were analyzed by Student’s unpaired t-tests or analysis of variance, as appropriate. P values ≤0.05 were considered significant. The details and analytical methods are indicated in the figure legends, Results or Methods sections. The immunohistochemically stained sections were scanned using an Aperio AT2 microscope (Leica Biosystems) at 20× magnification and analyzed with Aperio software. Quantification was performed by pathologists who were blinded to the diagnosis of the tissue cores. Flow cytometry analyzes were performed using BD FACSDiva software, and the results were analyzed using FlowJo 10.0 software. Western blot results were analyzed using ImageJ. The raw western blot data, PCR primers and supplementry methods can be found in the online supplemental files 1–3.

10.1136/jitc-2020-002138.supp1 Supplementary data

10.1136/jitc-2020-002138.supp2 Supplementary data

10.1136/jitc-2020-002138.supp3 Supplementary data
