# Supplementary figures and images for: N-cadherin inhibitor creates a microenvironment that protect TILs from immune checkpoints and Treg cells
Source: J Immunother Cancer. 2021 Mar 10;9(3):e002138. doi: 10.1136/jitc-2020-002138 (PMC7949480; doi:10.1136/jitc-2020-002138)

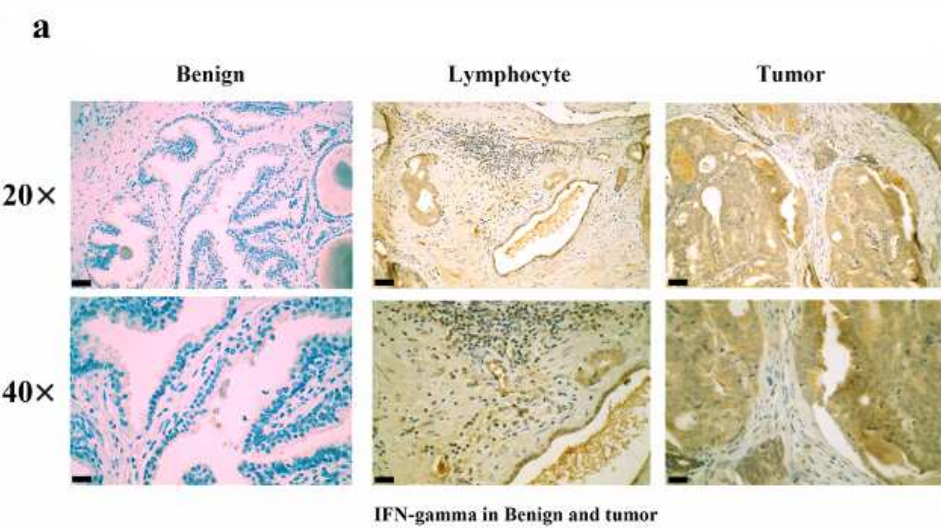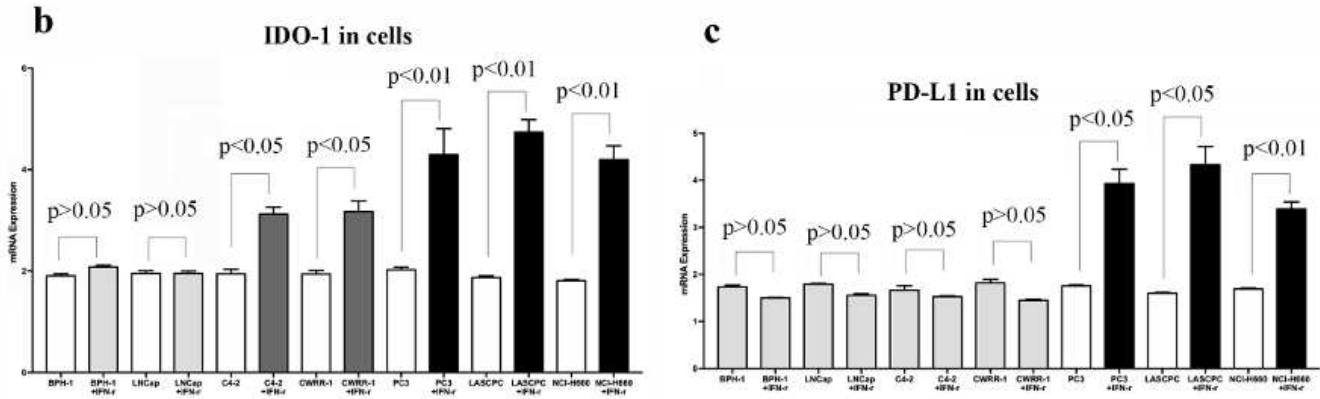

Supplement: Supplementary data [file jitc-2020-002138supp004.pdf]

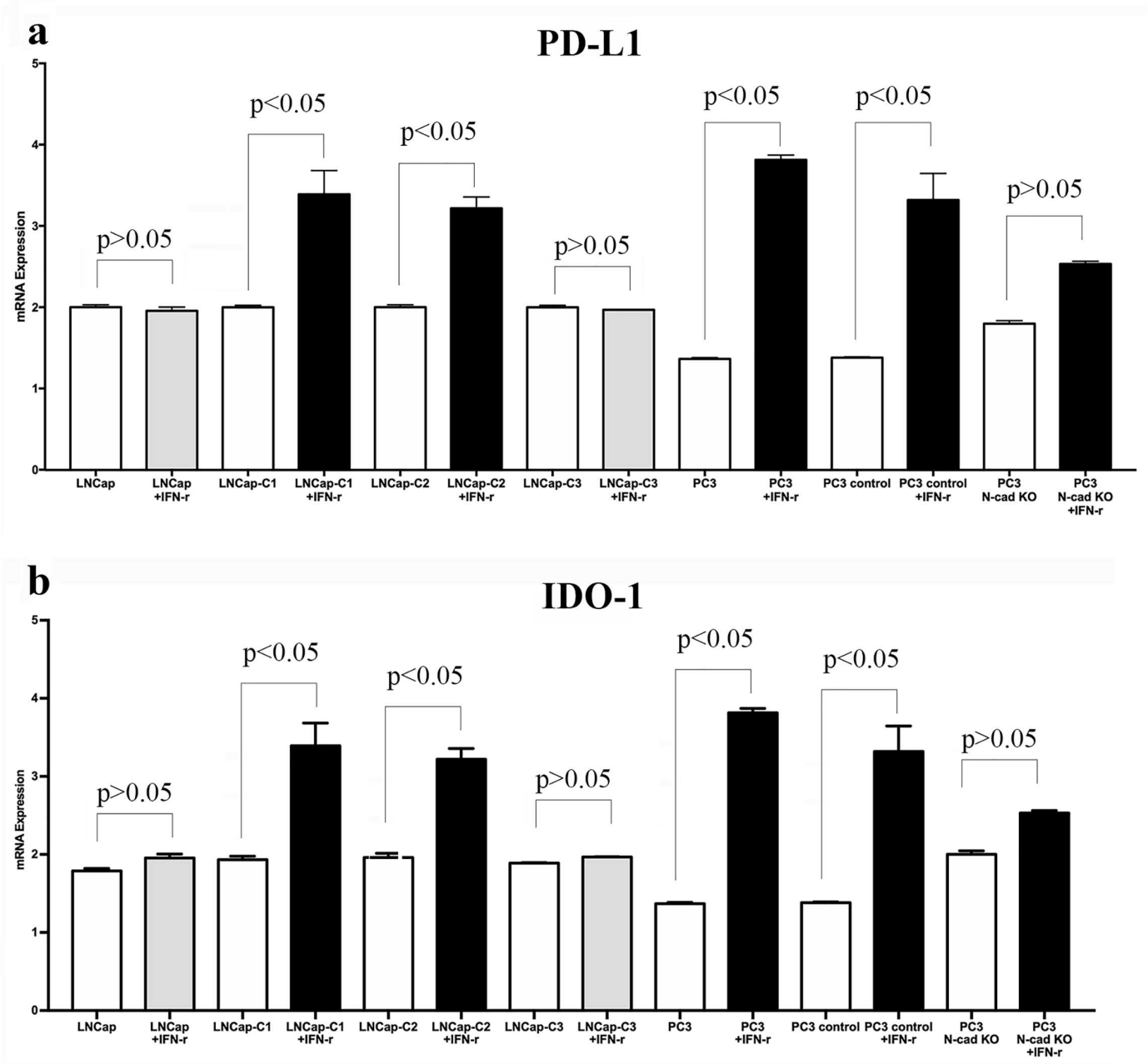

Supplement: Supplementary data [file jitc-2020-002138supp005.pdf]

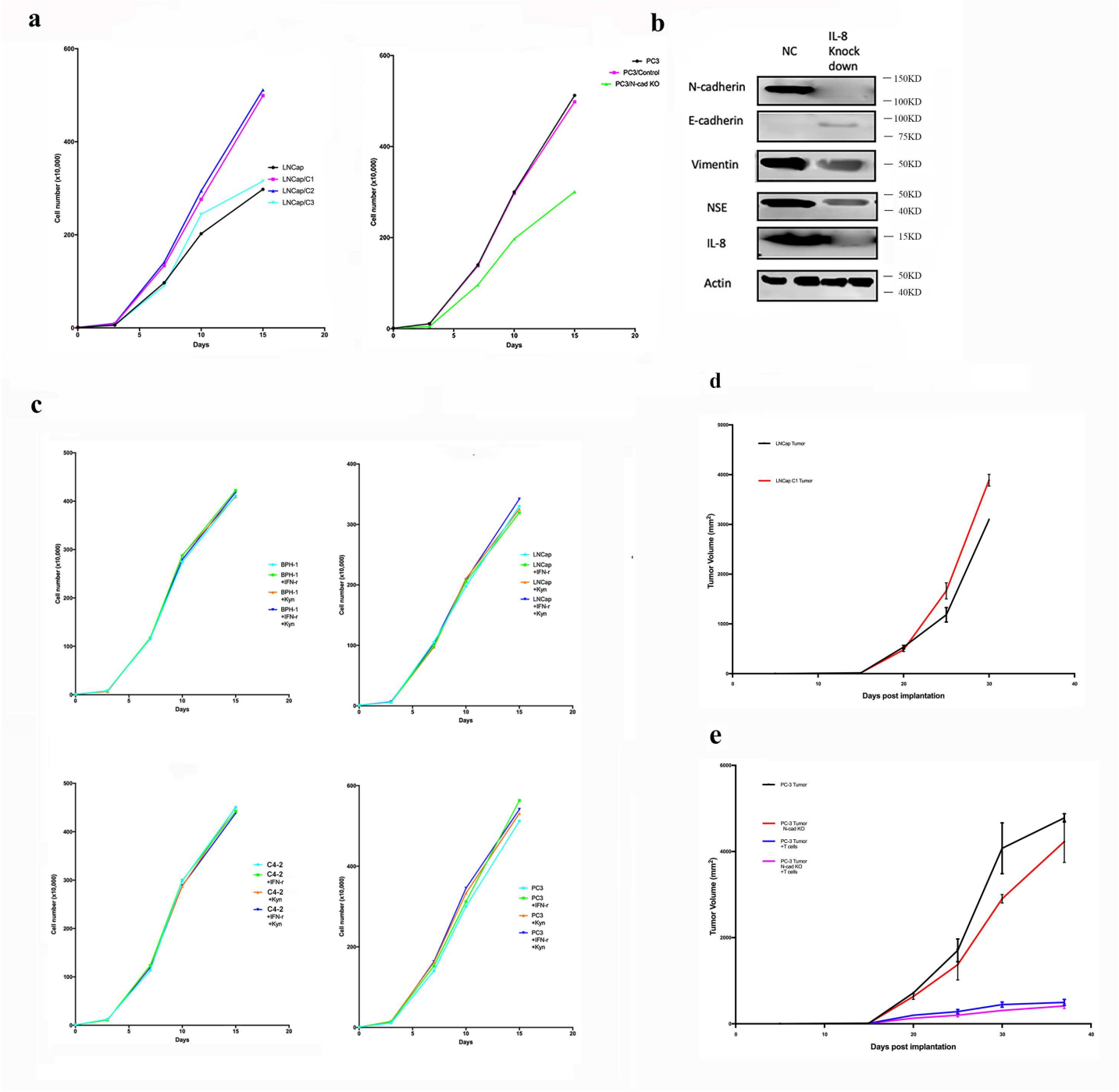

Supplement: Supplementary data [file jitc-2020-002138supp006.pdf]

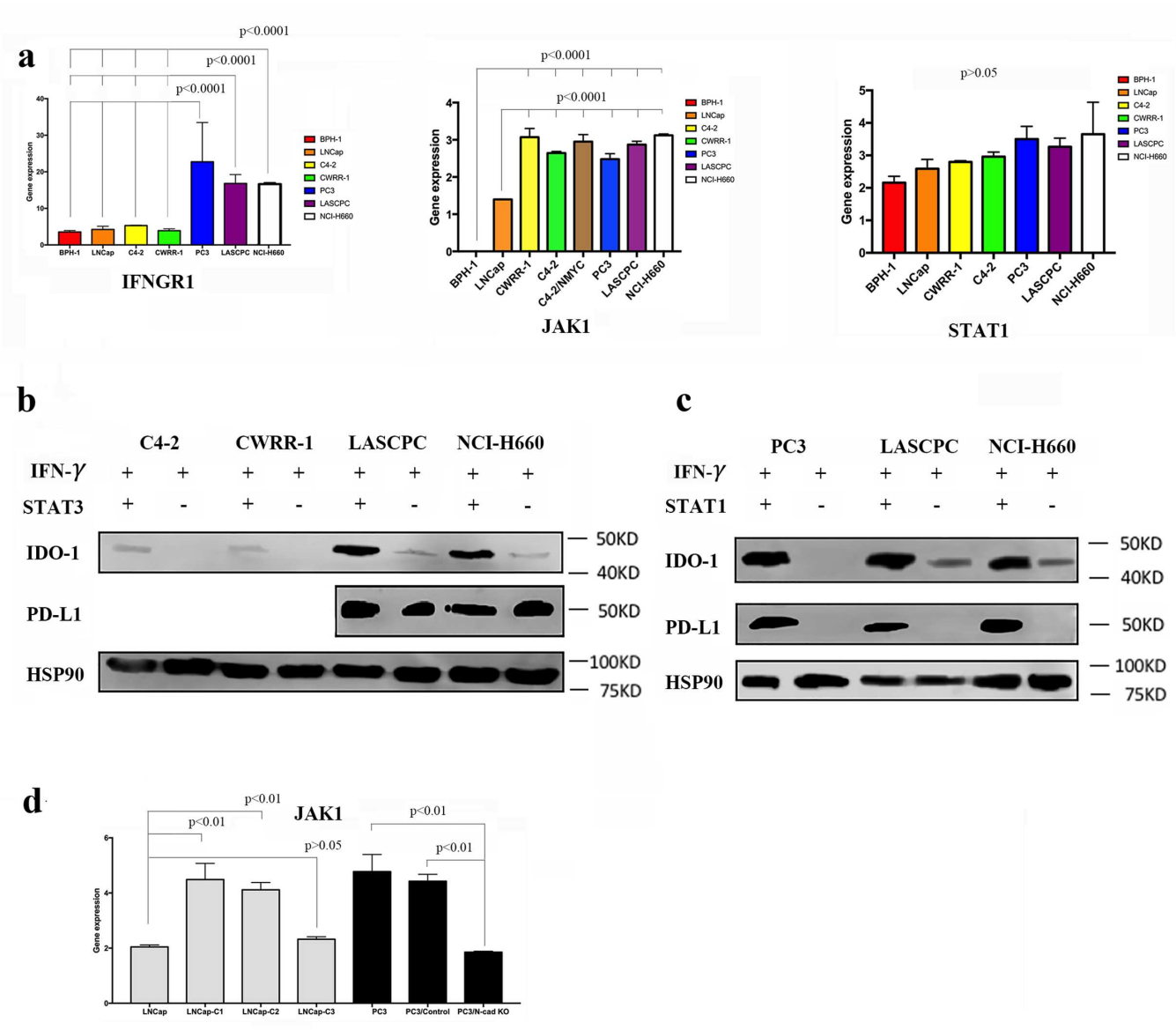

Supplement: Supplementary data [file jitc-2020-002138supp007.pdf]

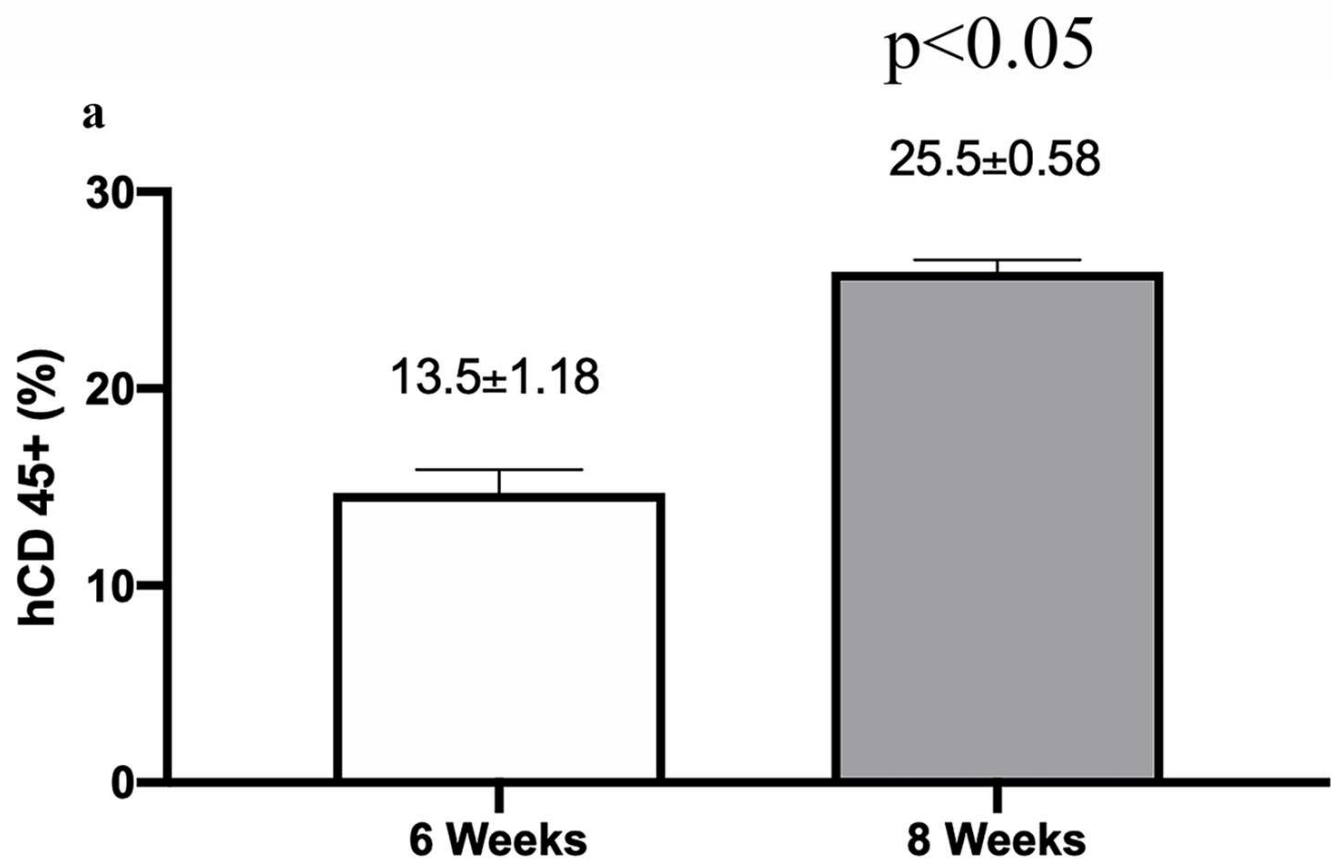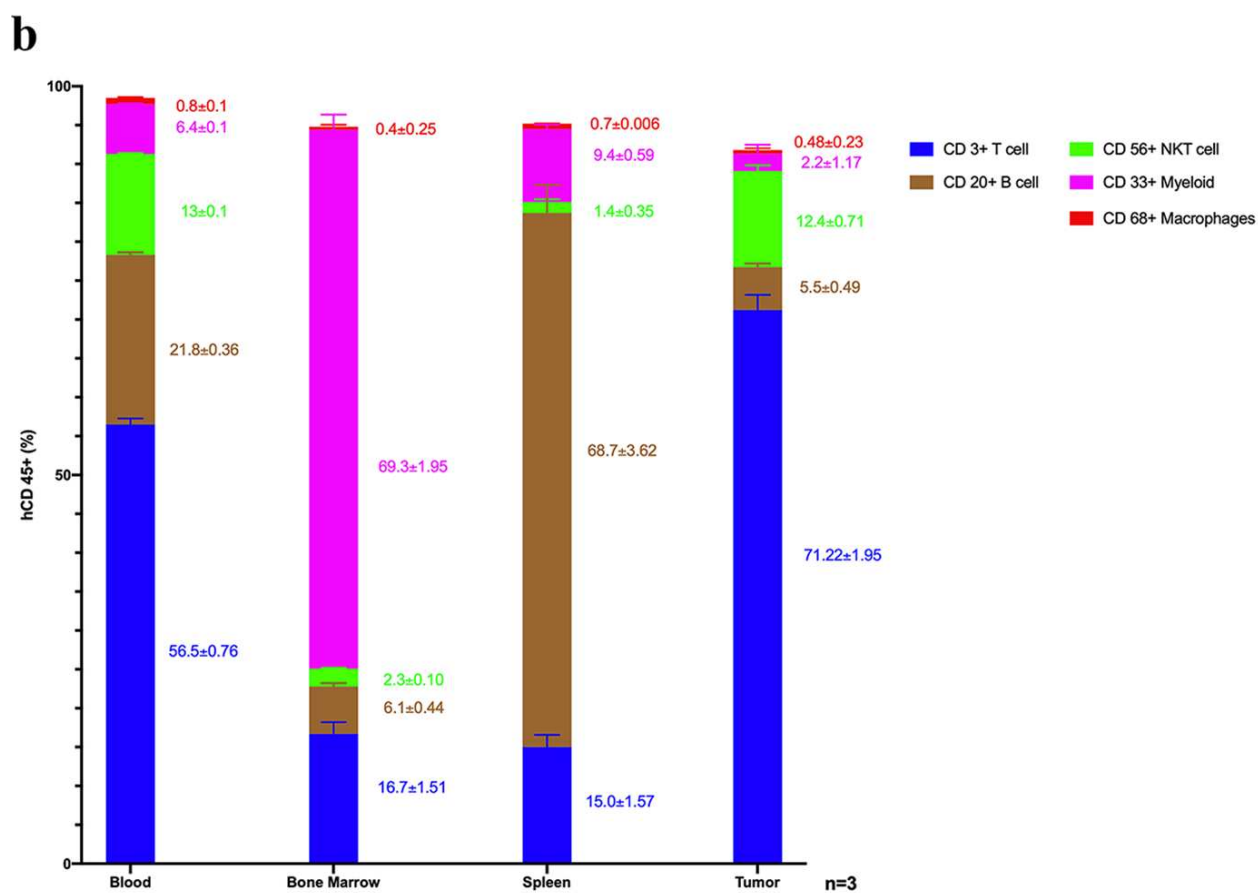

Supplement: Supplementary data [file jitc-2020-002138supp008.pdf]

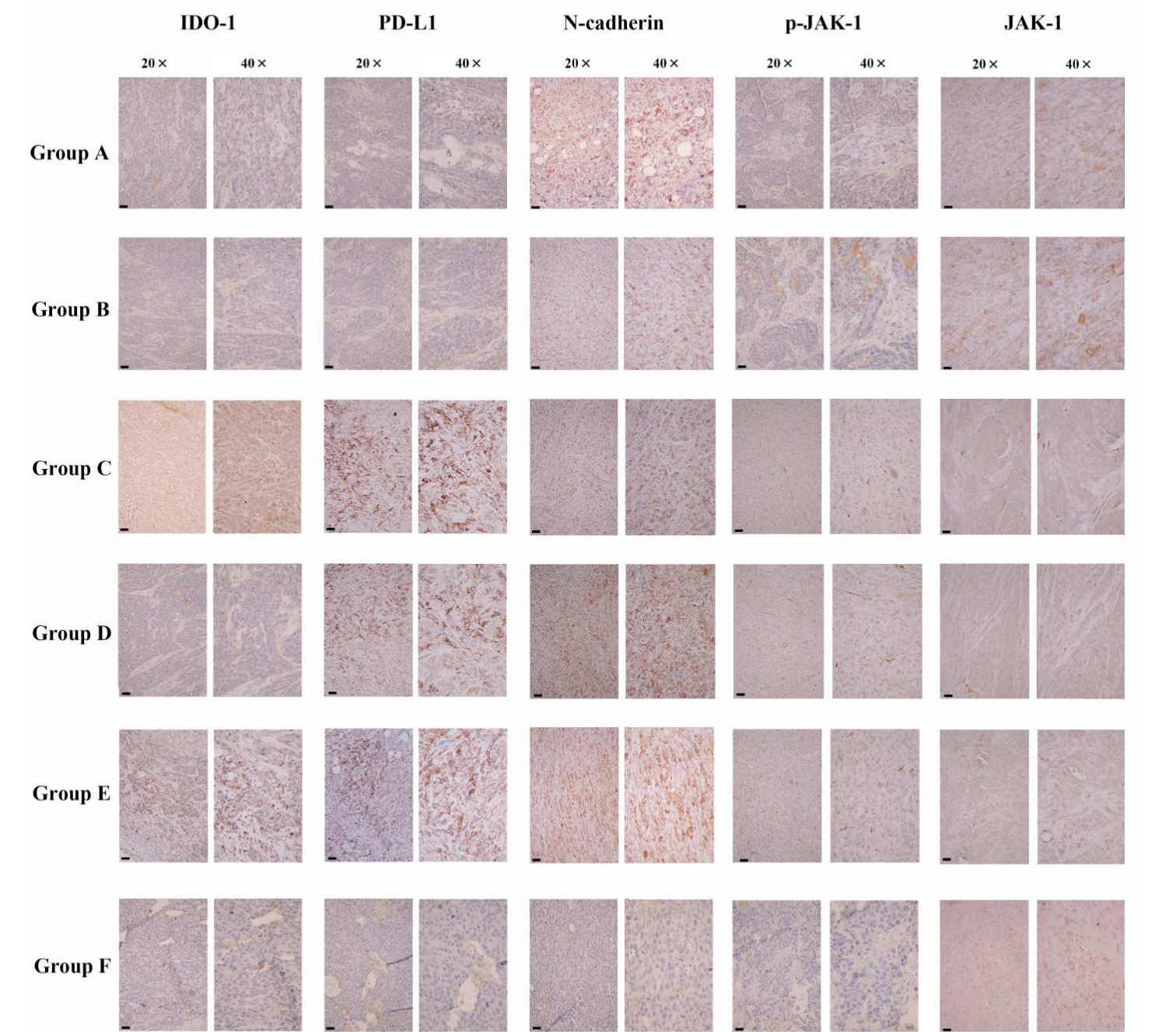

Supplement: Supplementary data [file jitc-2020-002138supp009.pdf]

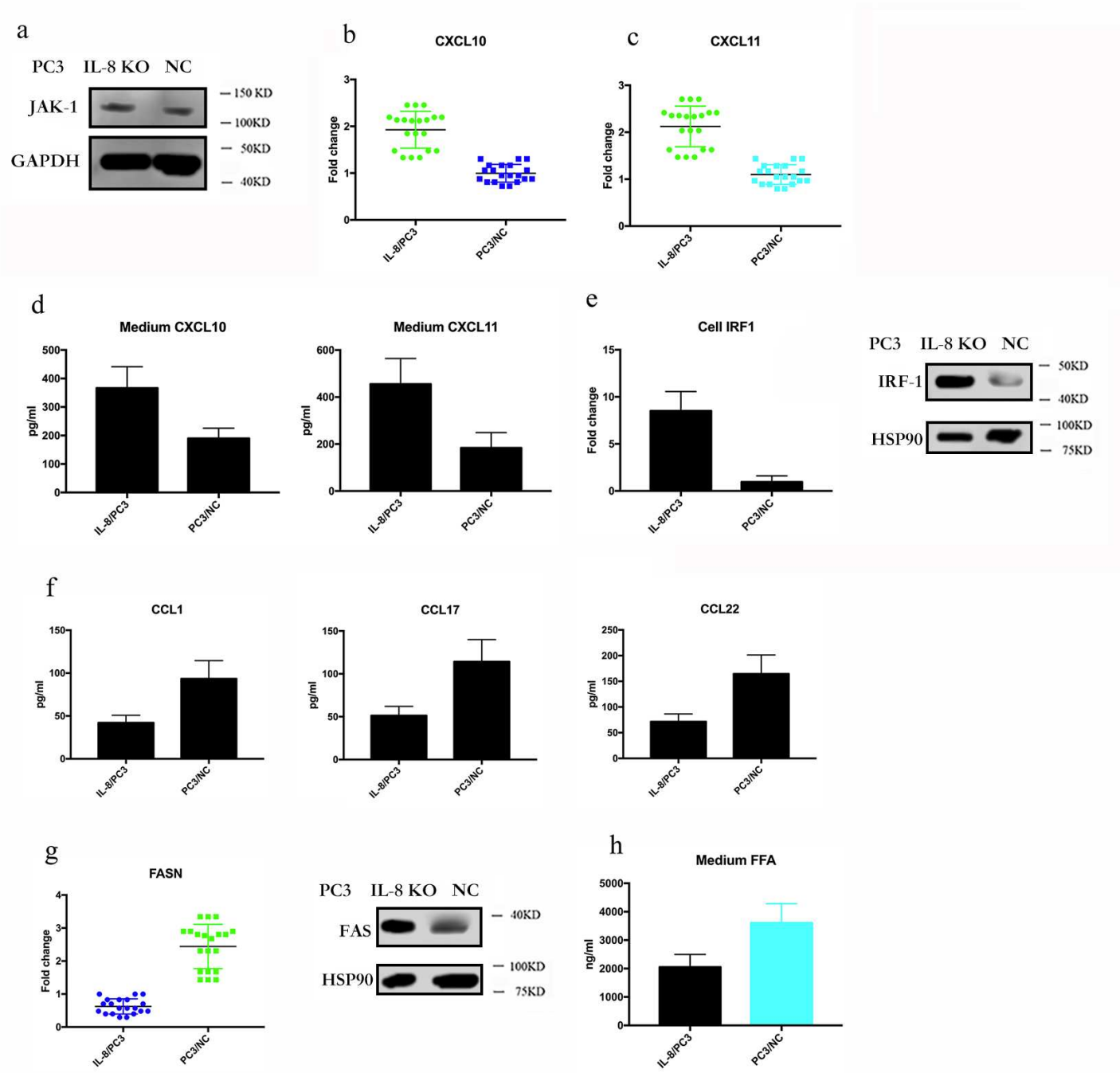

Supplement: Supplementary data [file jitc-2020-002138supp010.pdf]
