# Supplementary material for: N-cadherin inhibitor creates a microenvironment that protect TILs from immune checkpoints and Treg cells
Source: J Immunother Cancer. 2021 Mar 10;9(3):e002138. doi: 10.1136/jitc-2020-002138 (PMC7949480; doi:10.1136/jitc-2020-002138)

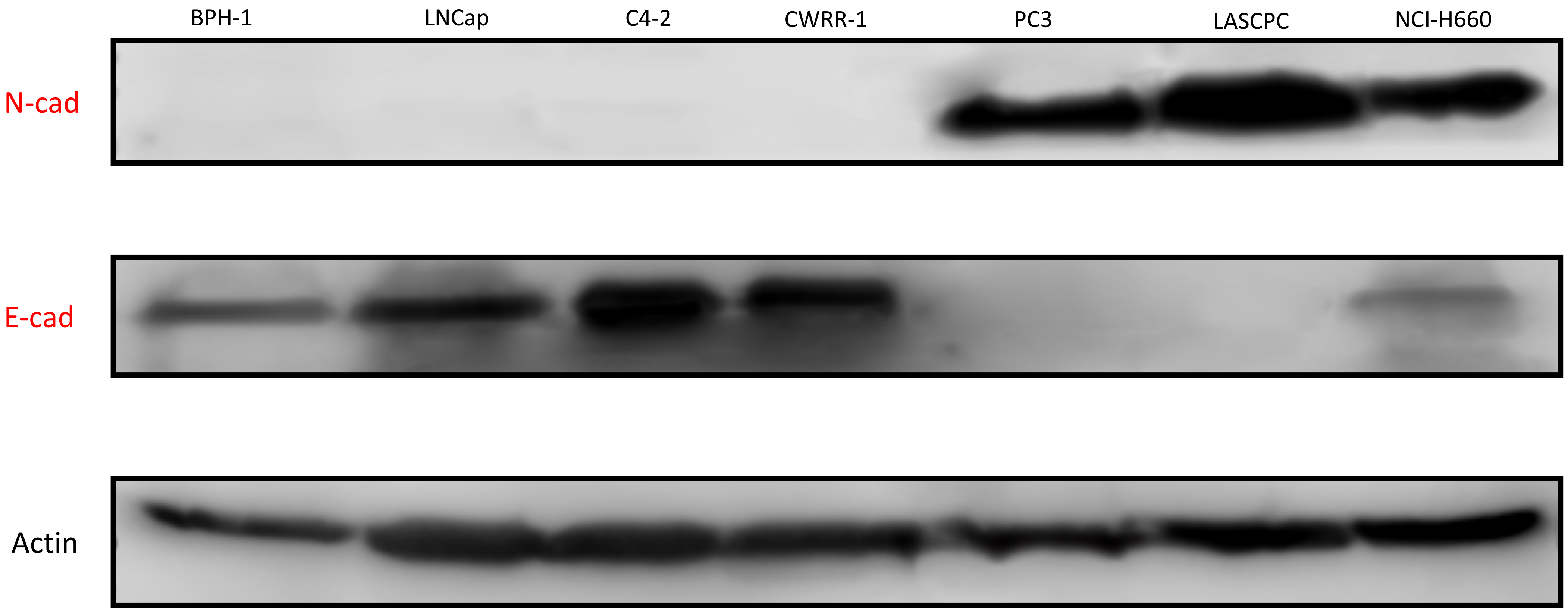
BPH-1
LNCap
C4-2
CWRR-1
PC3
LASCPC
NCI-H660
N-cad
E-cad
Actin

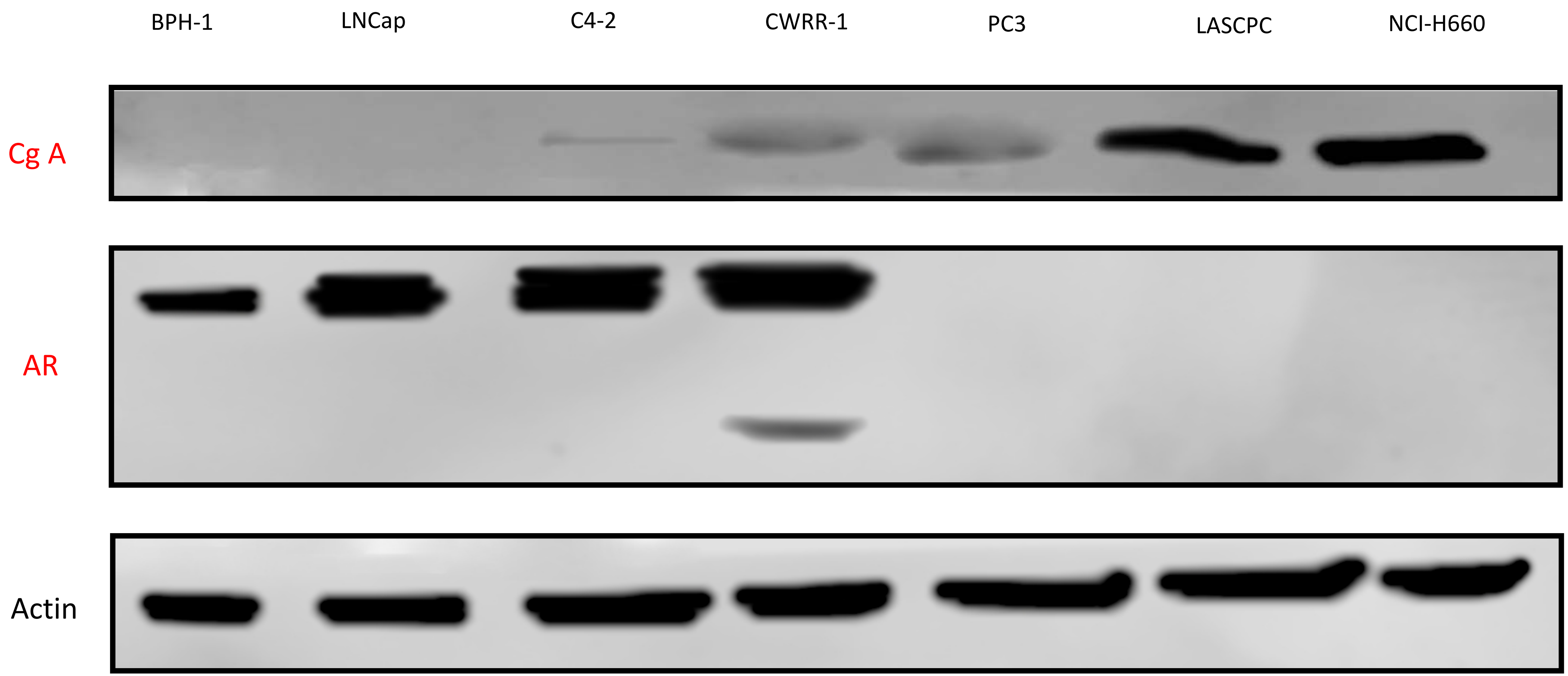
BPH-1
LNCap
C4-2
CWRR-1
PC3
LASCPC
NCI-H660
Cg A
AR
Actin

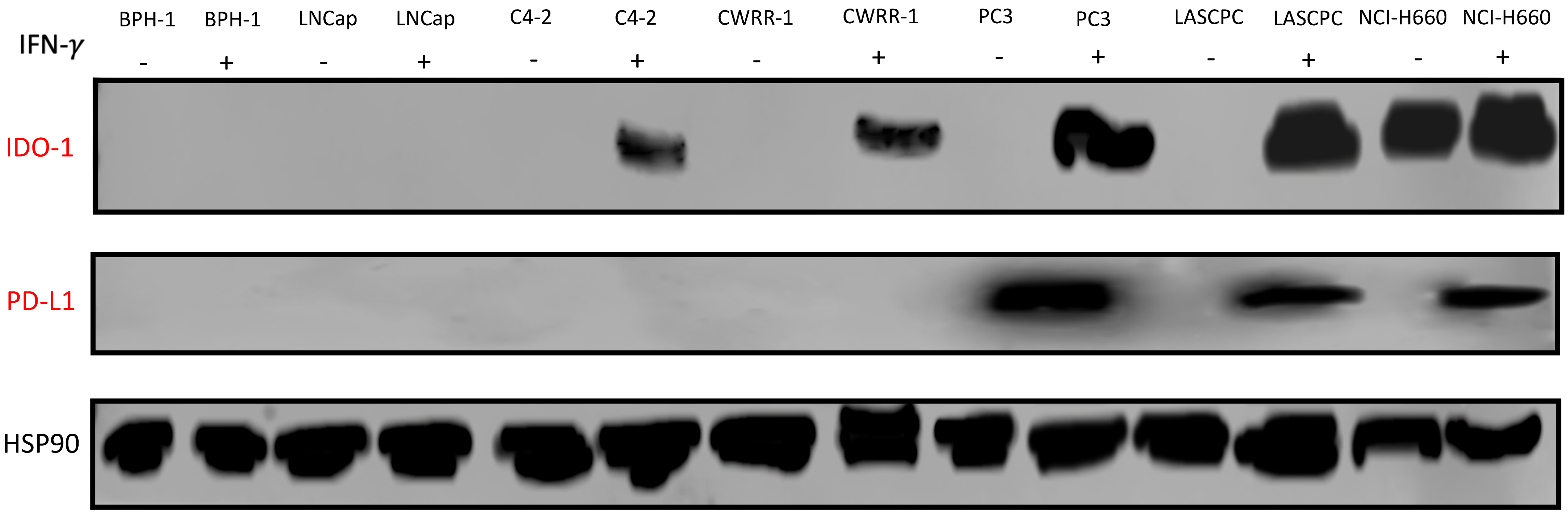
BPH-1 BPH-1 LNCap LNCap C4-2 C4-2 CWRR-1 CWRR-1 PC3 PC3 LASCPC LASCPC NCI-H660 NCI-H660
IFN-γ
- + - + - + - + - + - + - +
IDO-1
PD-L1
HSP90

BMJ Publishing Group Limited (BMJ) disclaims all liability and responsibility arising from any reliance placed on this supplemental material which has been supplied by the author(s)

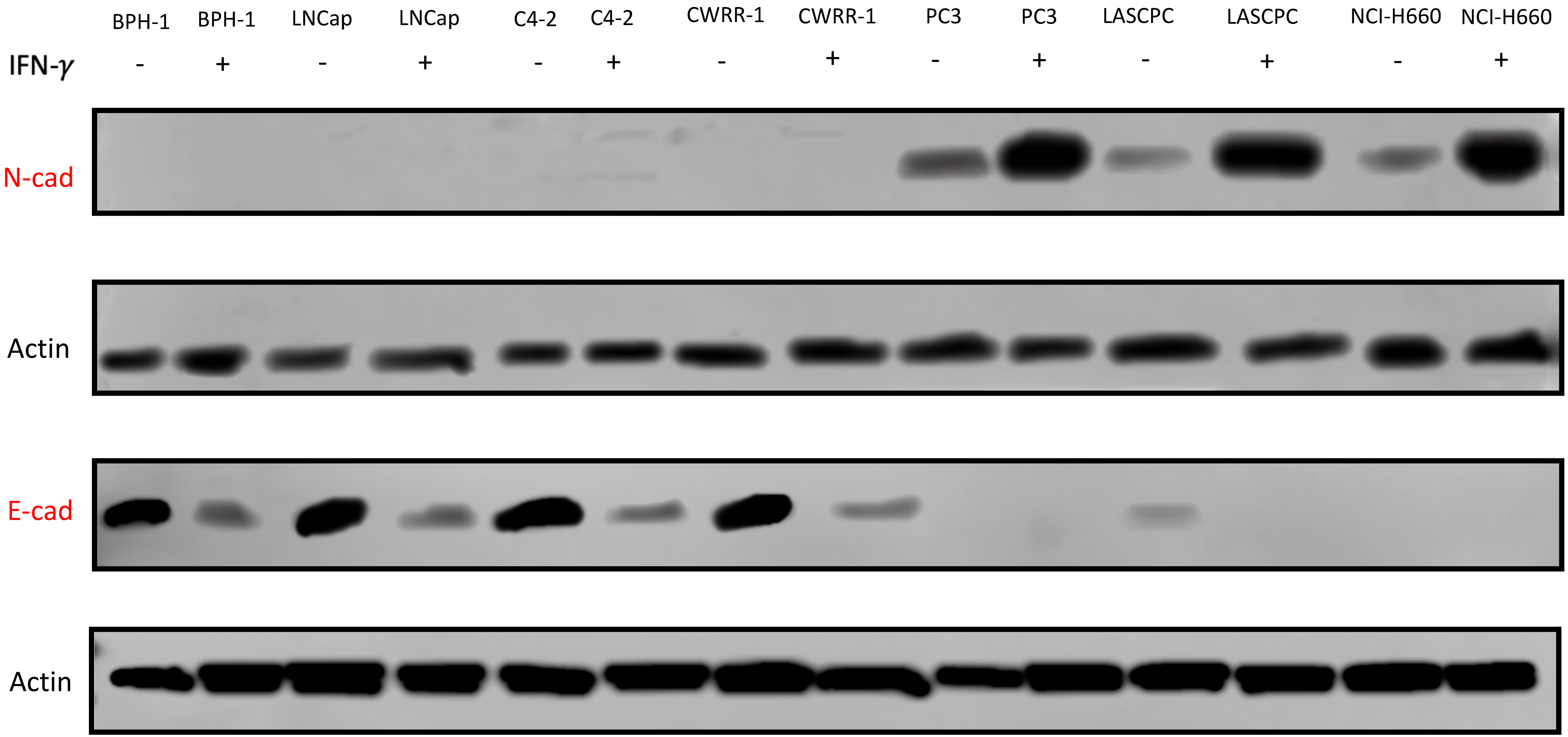

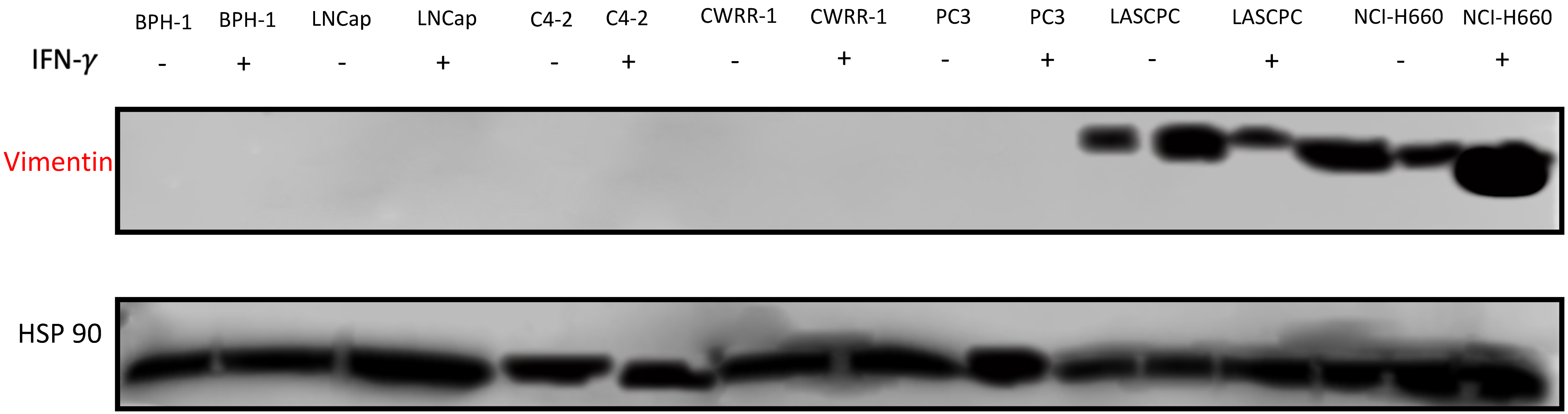
BPH-1 BPH-1 LNCap LNCap C4-2 C4-2 CWRR-1 CWRR-1 PC3 PC3 LASCPC LASCPC NCI-H660 NCI-H660
IFN-γ - + - + - + - + - + - + - +
Vimentin
HSP 90

BMJ Publishing Group Limited (BMJ) disclaims all liability and responsibility arising from any reliance placed on this supplemental material which has been supplied by the author(s)

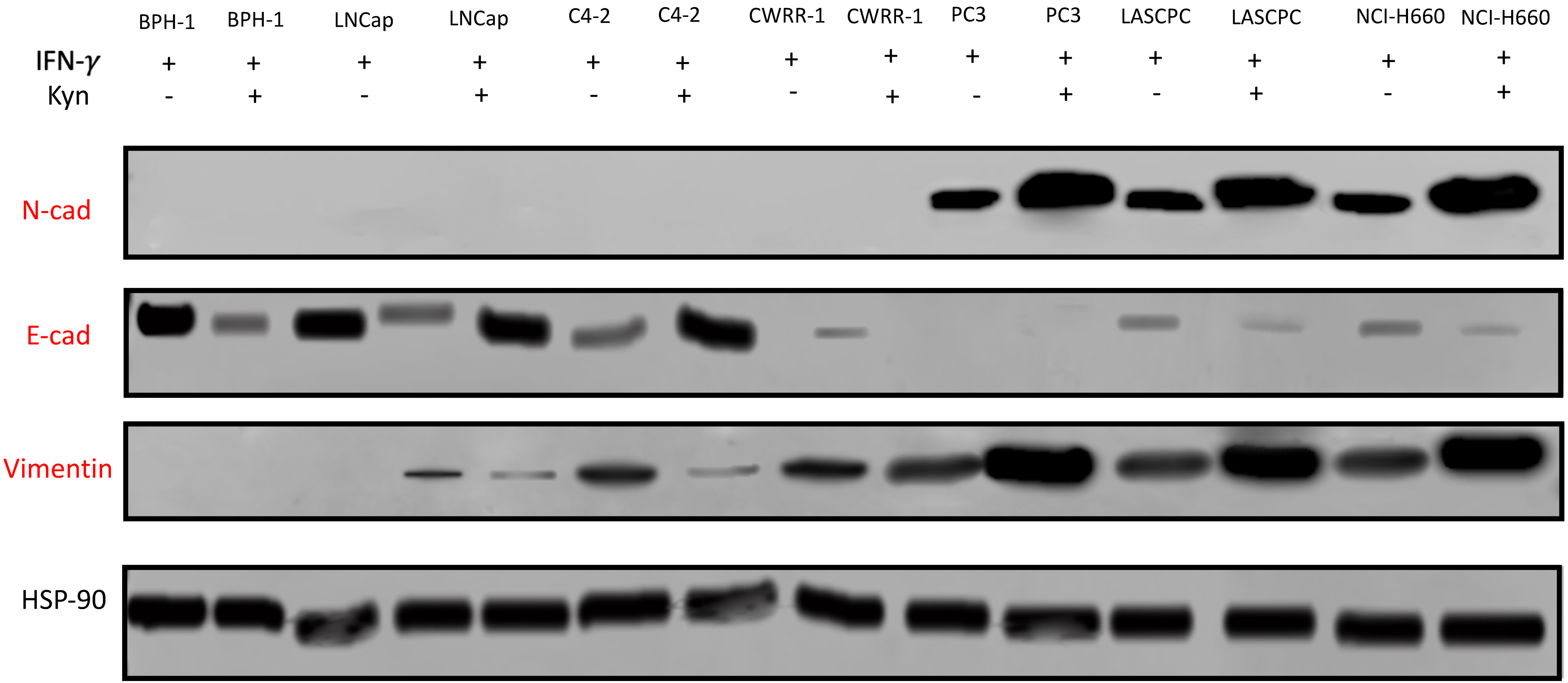

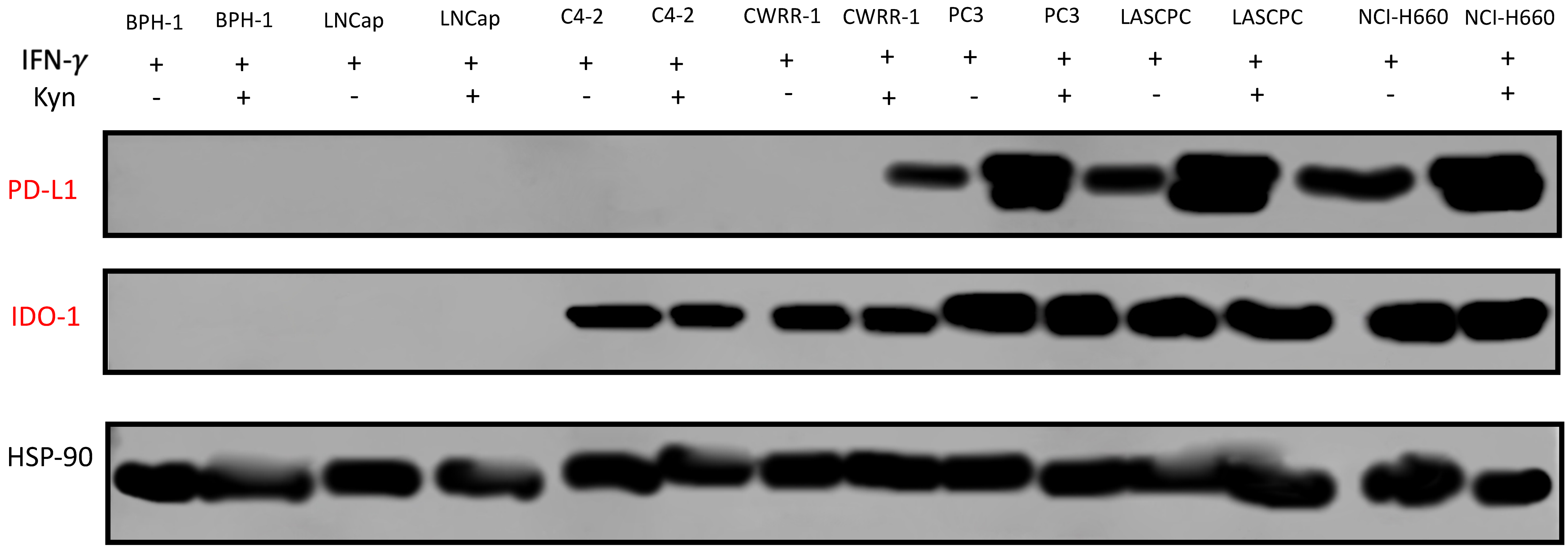
BPH-1
BPH-1
LNCap
LNCap
C4-2
C4-2
CWRR-1
CWRR-1
PC3
PC3
LASCPC
LASCPC
NCI-H660
NCI-H660
IFN-γ
Kyn
PD-L1
IDO-1
HSP-90

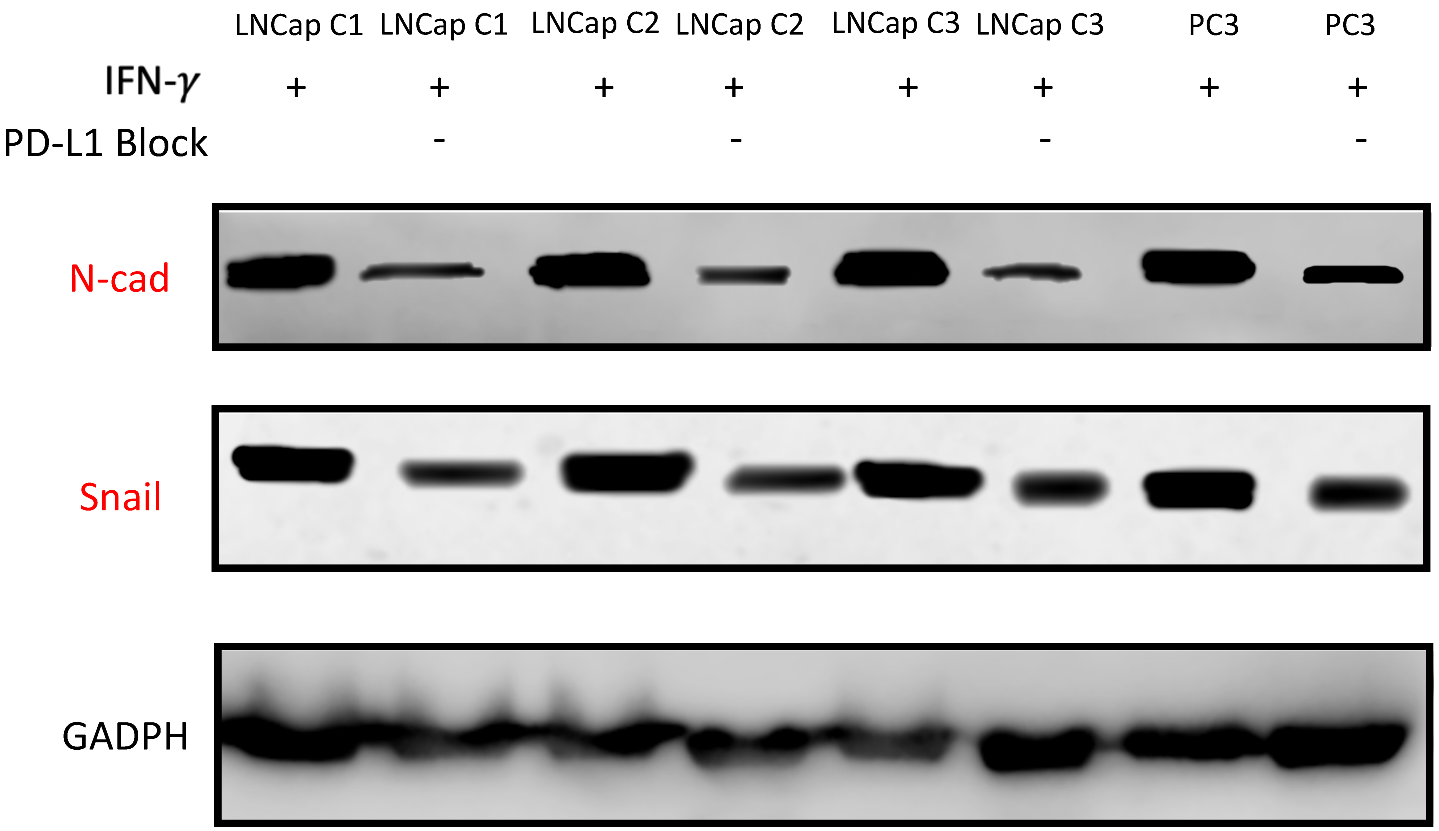
LNCap C1
LNCap C1
LNCap C2
LNCap C2
LNCap C3
LNCap C3
PC3
PC3
IFN-γ
+ + + + + + + +
PD-L1 Block
- - - -
N-cad
Snail
GADPH

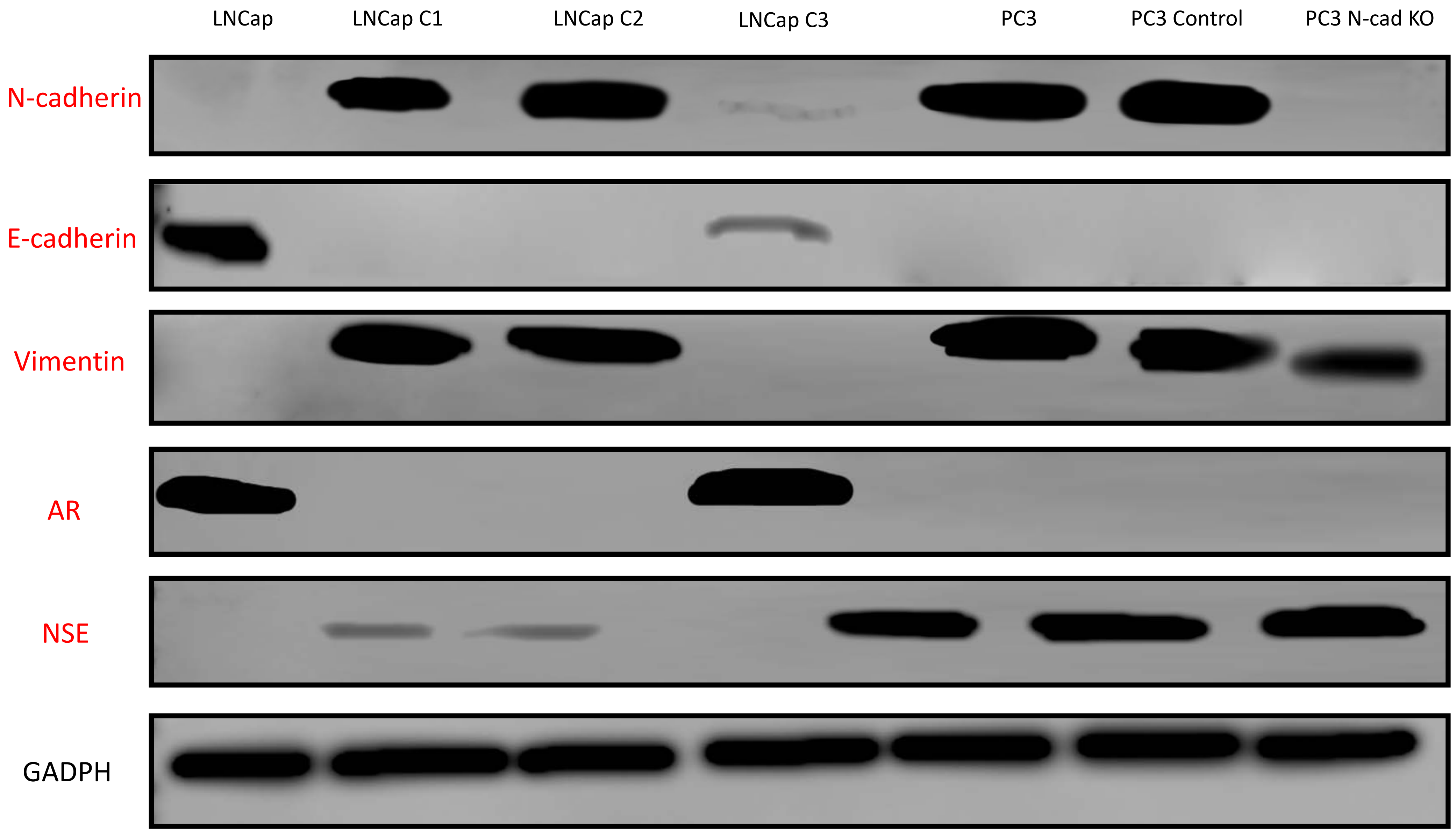
LNCap
LNCap C1
LNCap C2
LNCap C3
PC3
PC3 Control
PC3 N-cad KO
N-cadherin
E-cadherin
Vimentin
AR
NSE
GADPH

BMJ Publishing Group Limited (BMJ) disclaims all liability and responsibility arising from any reliance
placed on this supplemental material which has been supplied by the author(s)

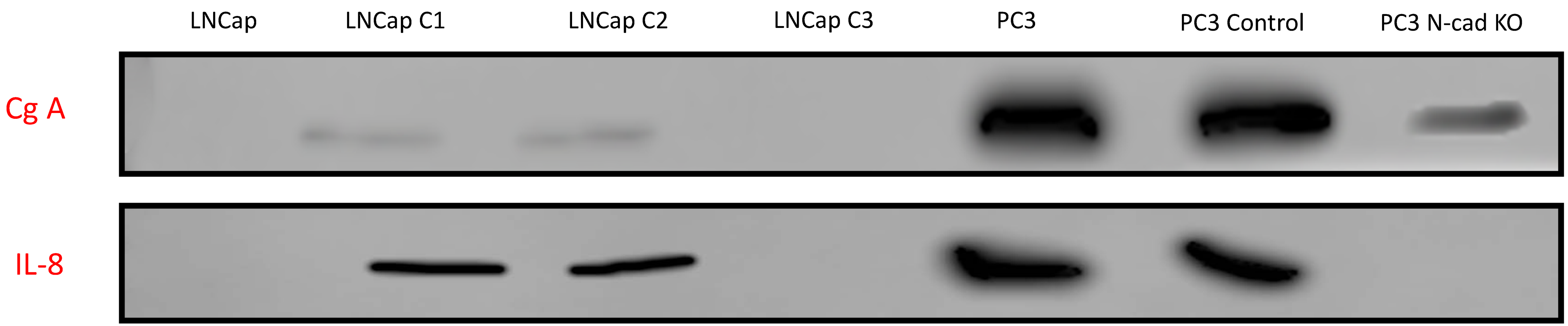

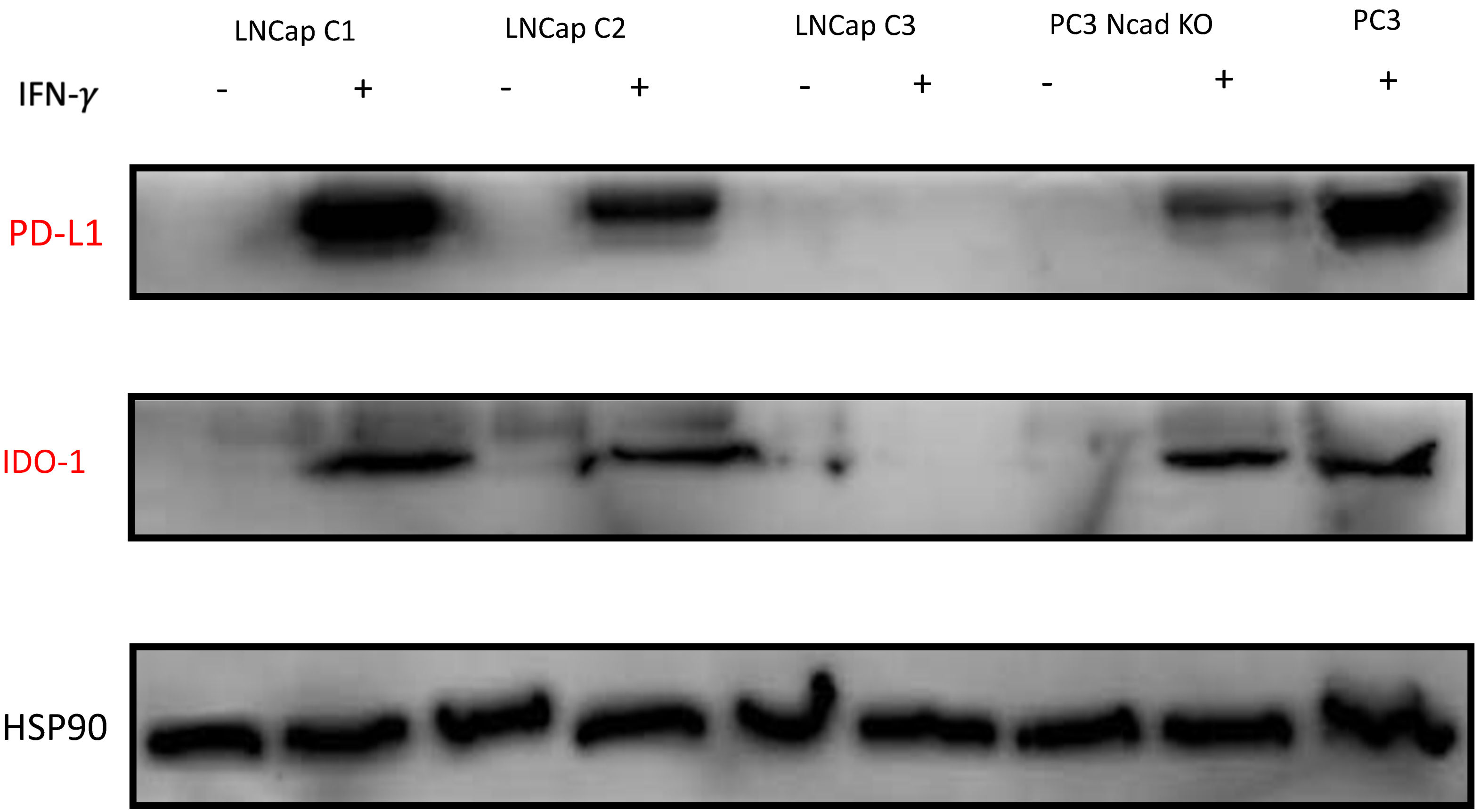
LNCap C1
LNCap C2
LNCap C3
PC3 Ncad KO
PC3
IFN-γ
- + - + - + - + +
PD-L1
IDO-1
HSP90

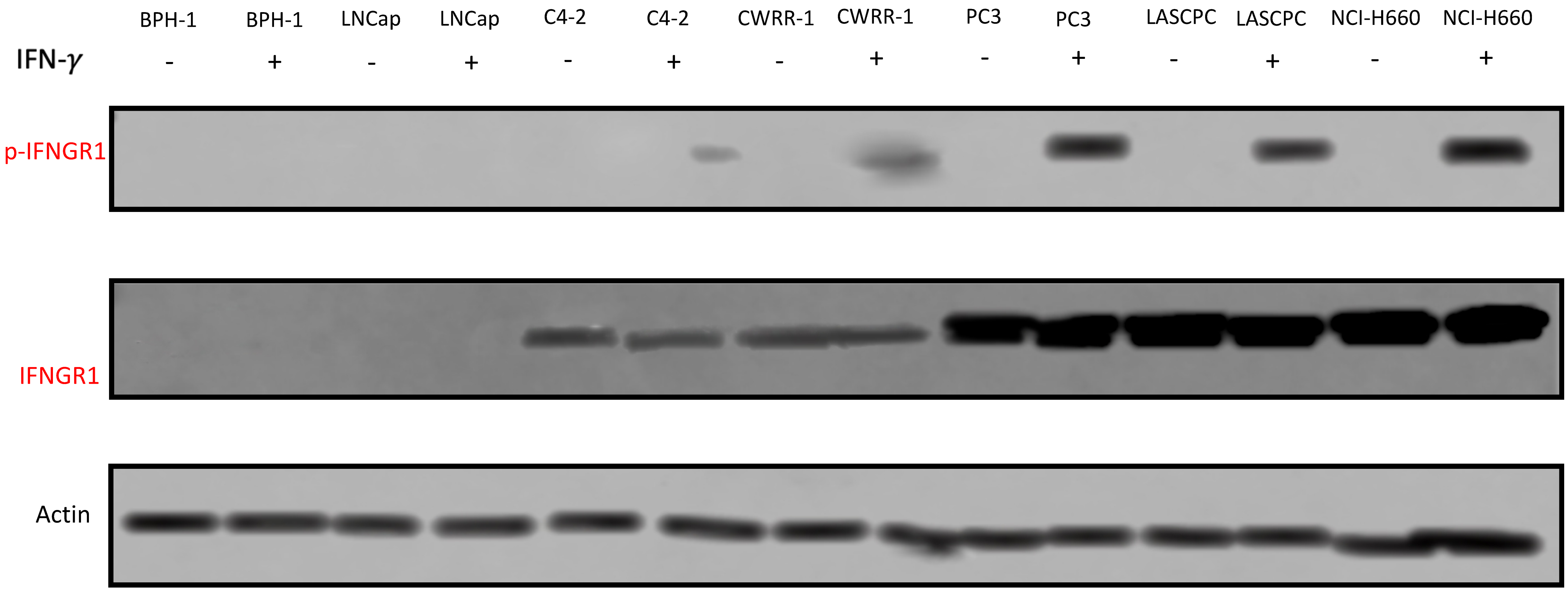
BPH-1
BPH-1
LNCap
LNCap
C4-2
C4-2
CWRR-1
CWRR-1
PC3
PC3
LASCPC
LASCPC
NCI-H660
NCI-H660
IFN-γ
- + - + - + - + - + - + - +
p-IFNGR1
IFNGR1
Actin

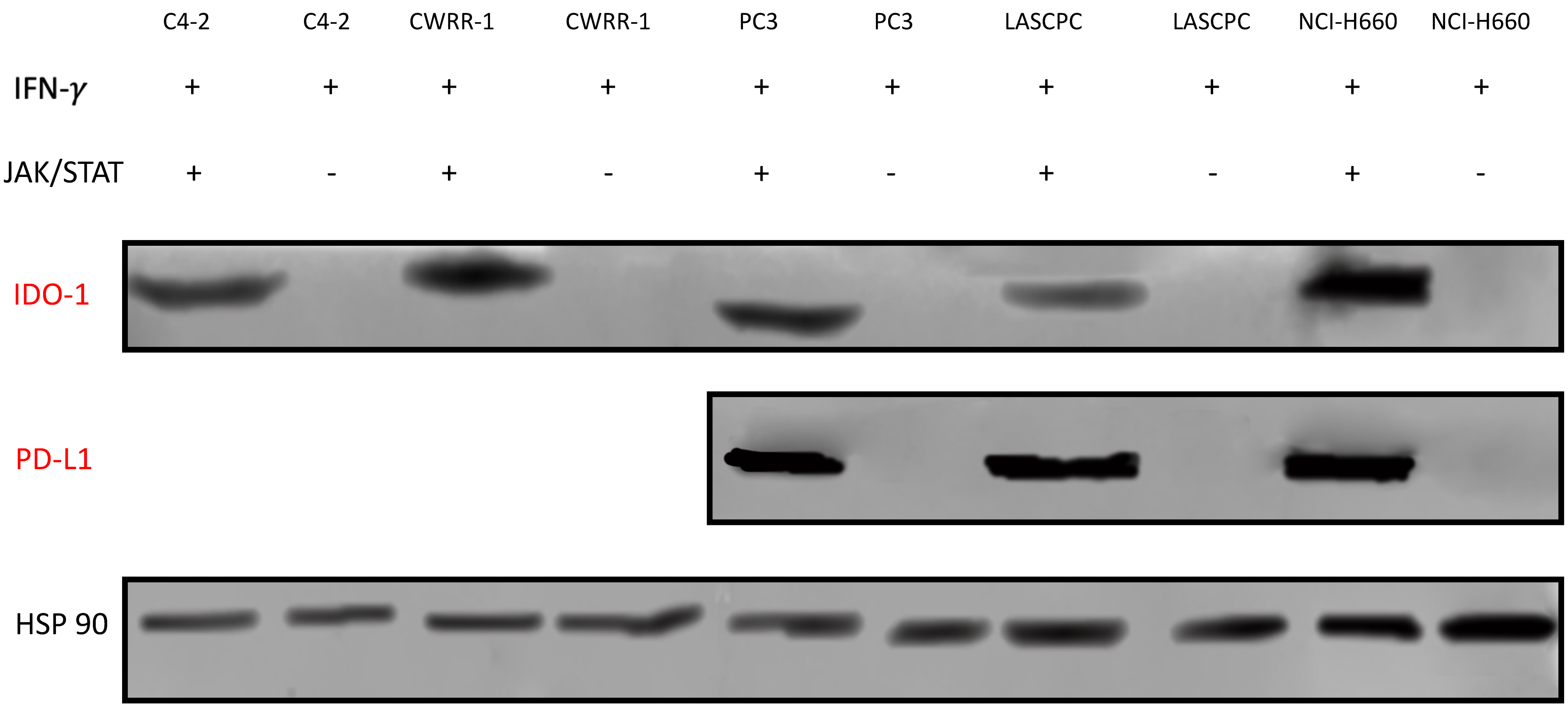
C4-2
C4-2
CWRR-1
CWRR-1
PC3
PC3
LASCPC
LASCPC
NCI-H660
NCI-H660
IFN-γ + + + + + + + + + +
JAK/STAT + - + - + - + - + -
IDO-1
PD-L1
HSP 90

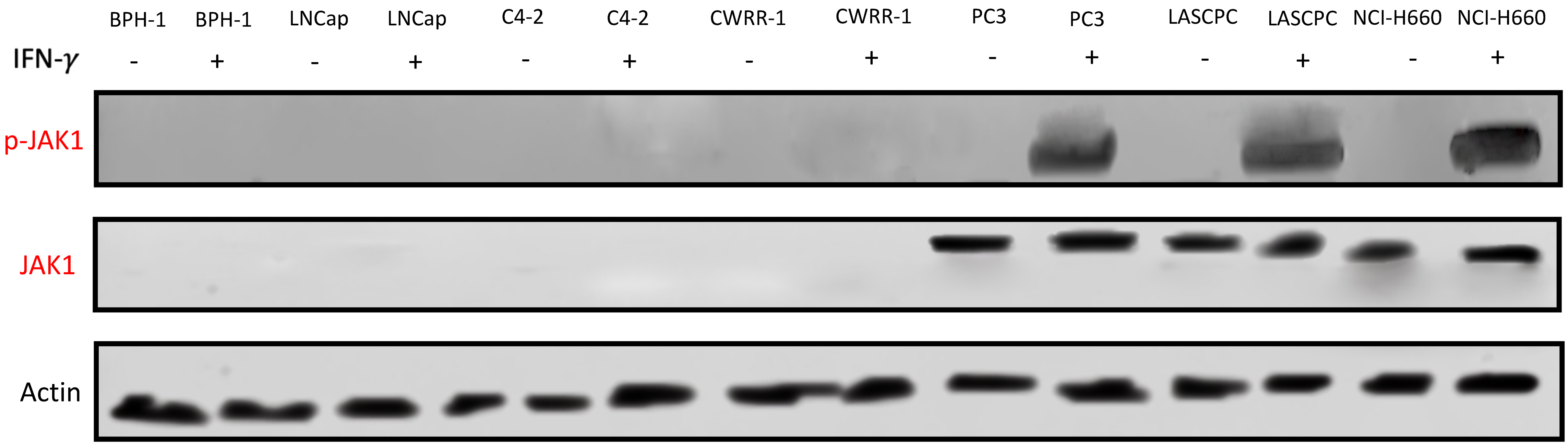
BPH-1
BPH-1
LNCap
LNCap
C4-2
C4-2
CWRR-1
CWRR-1
PC3
PC3
LASCPC
LASCPC
NCI-H660
NCI-H660
IFN-γ
- + - + - + - + - + - + - +
p-JAK1
JAK1
Actin

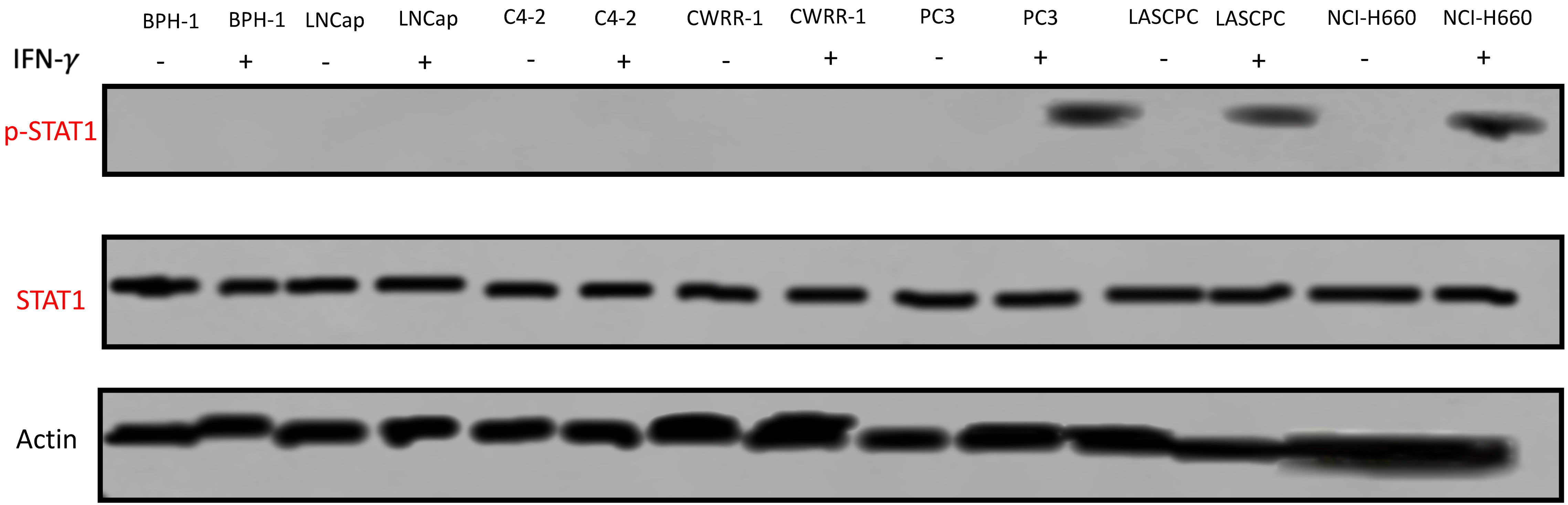
BPH-1
BPH-1
LNCap
LNCap
C4-2
C4-2
CWRR-1
CWRR-1
PC3
PC3
LASCPC
LASCPC
NCI-H660
NCI-H660
IFN-γ
- + - + - + - + - + - + - +
p-STAT1
STAT1
Actin

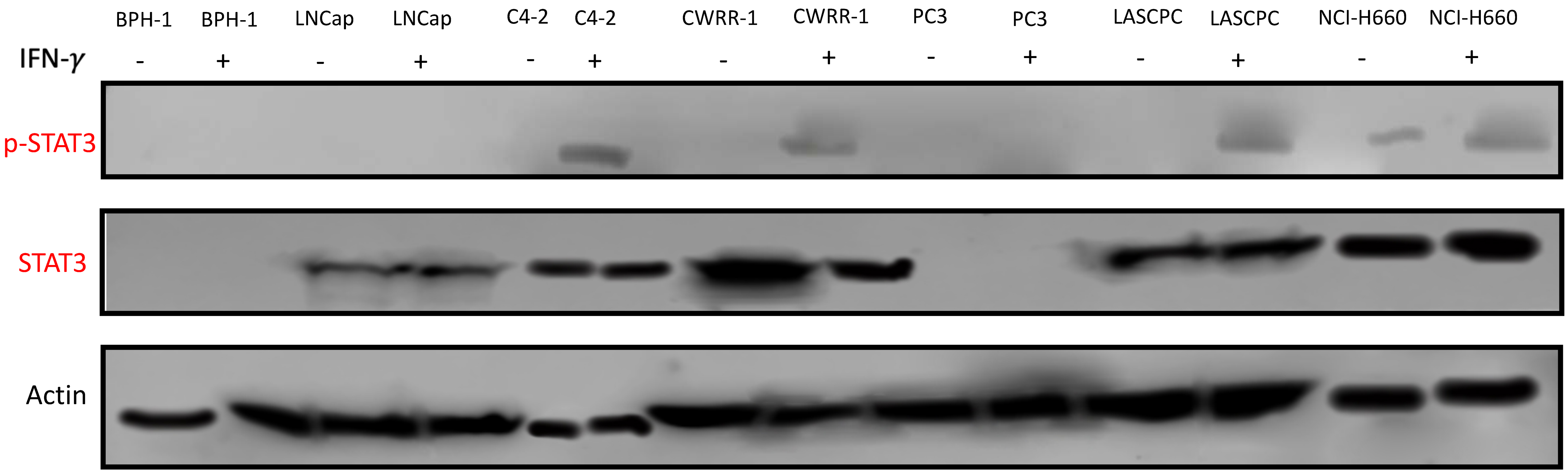
BPH-1
BPH-1
LNCap
LNCap
C4-2
C4-2
CWRR-1
CWRR-1
PC3
PC3
LASCPC
LASCPC
NCI-H660
NCI-H660
IFN-γ
- + - + - + - + - + - + - +
p-STAT3
STAT3
Actin

BMJ Publishing Group Limited (BMJ) disclaims all liability and responsibility arising from any reliance placed on this supplemental material which has been supplied by the author(s)

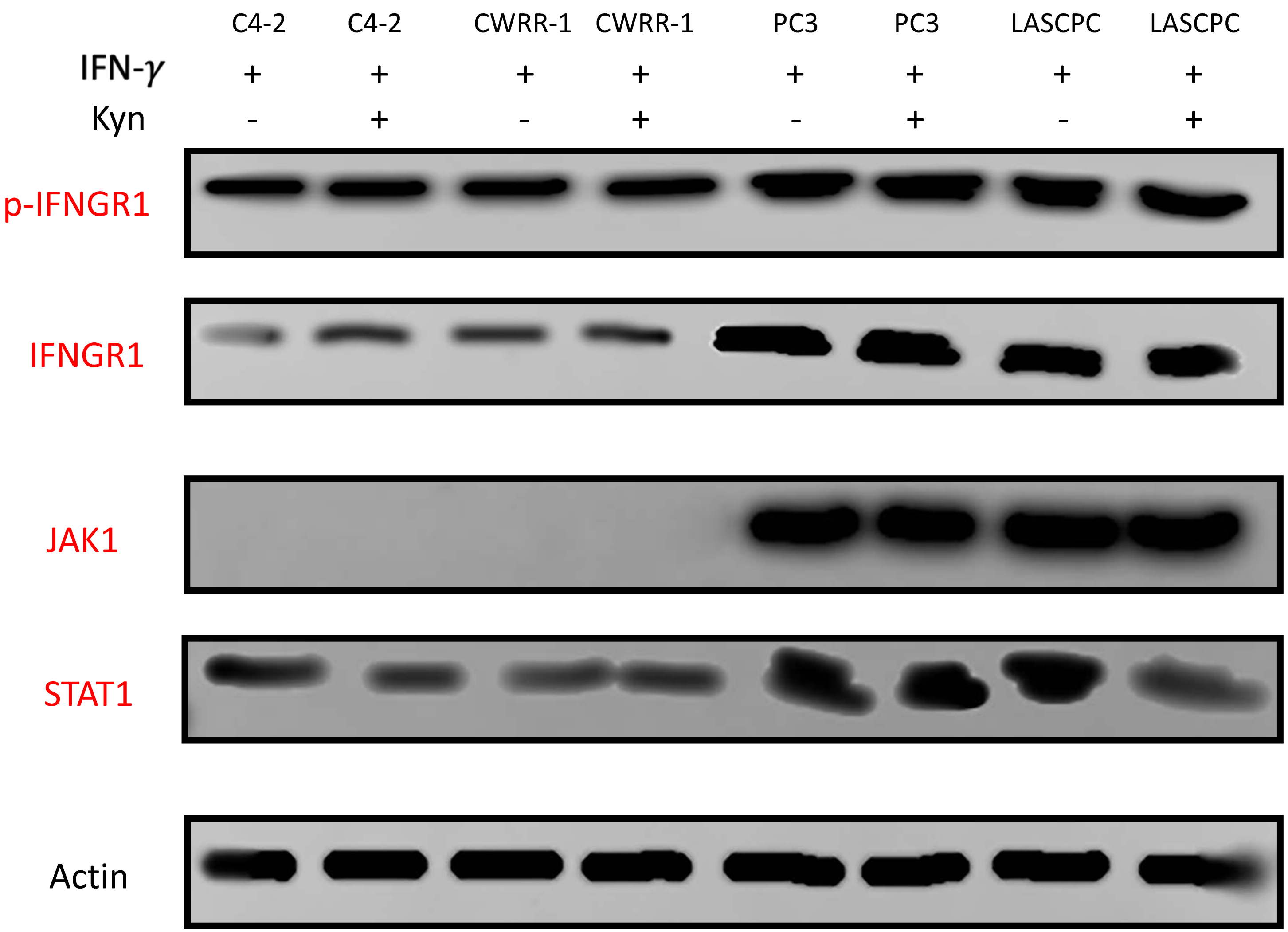

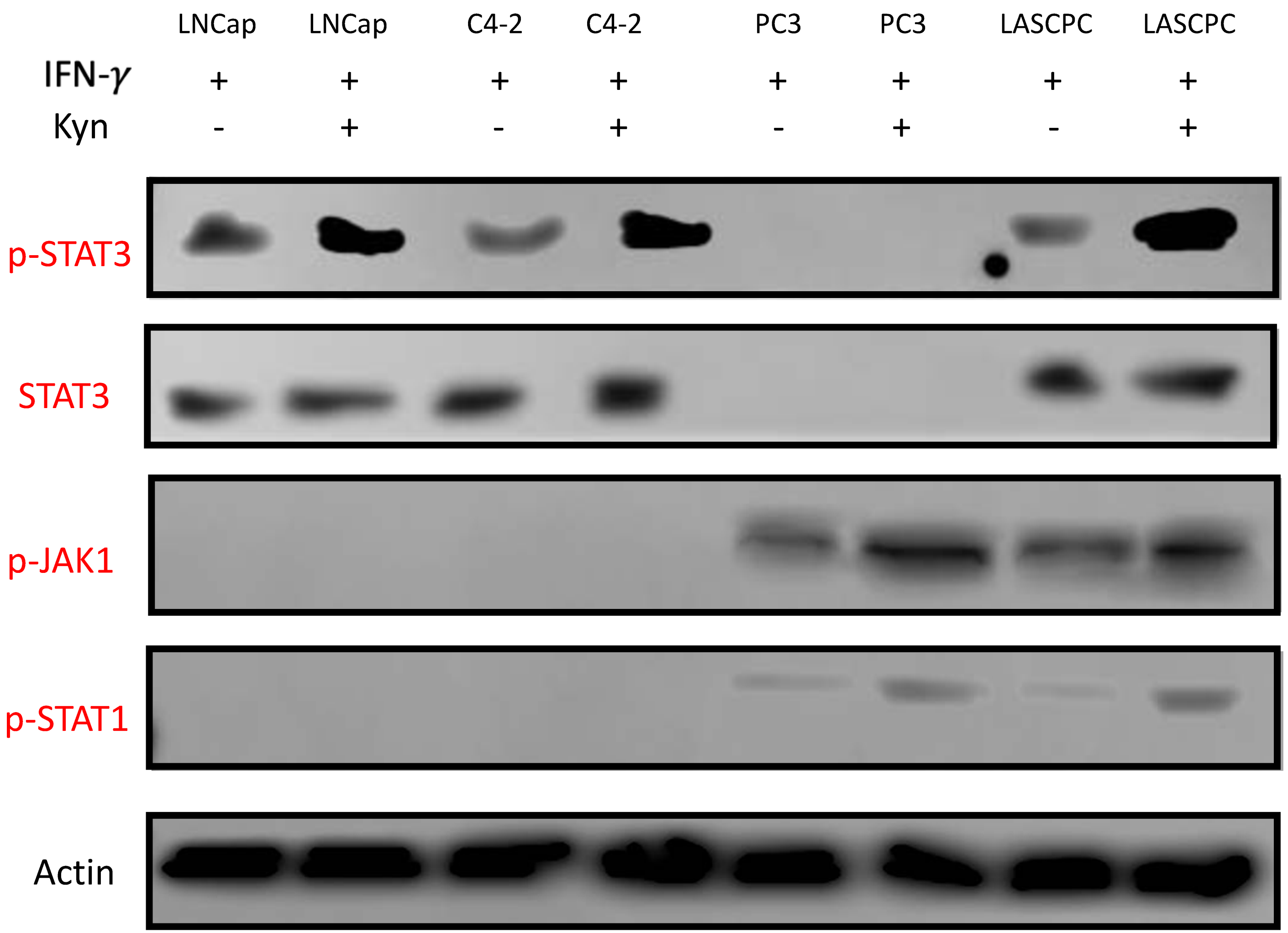
LNCap
LNCap
C4-2
C4-2
PC3
PC3
LASCPC
LASCPC
IFN-γ
+ + + + + + + +
Kyn
- + - + - + - +
p-STAT3
STAT3
p-JAK1
p-STAT1
Actin

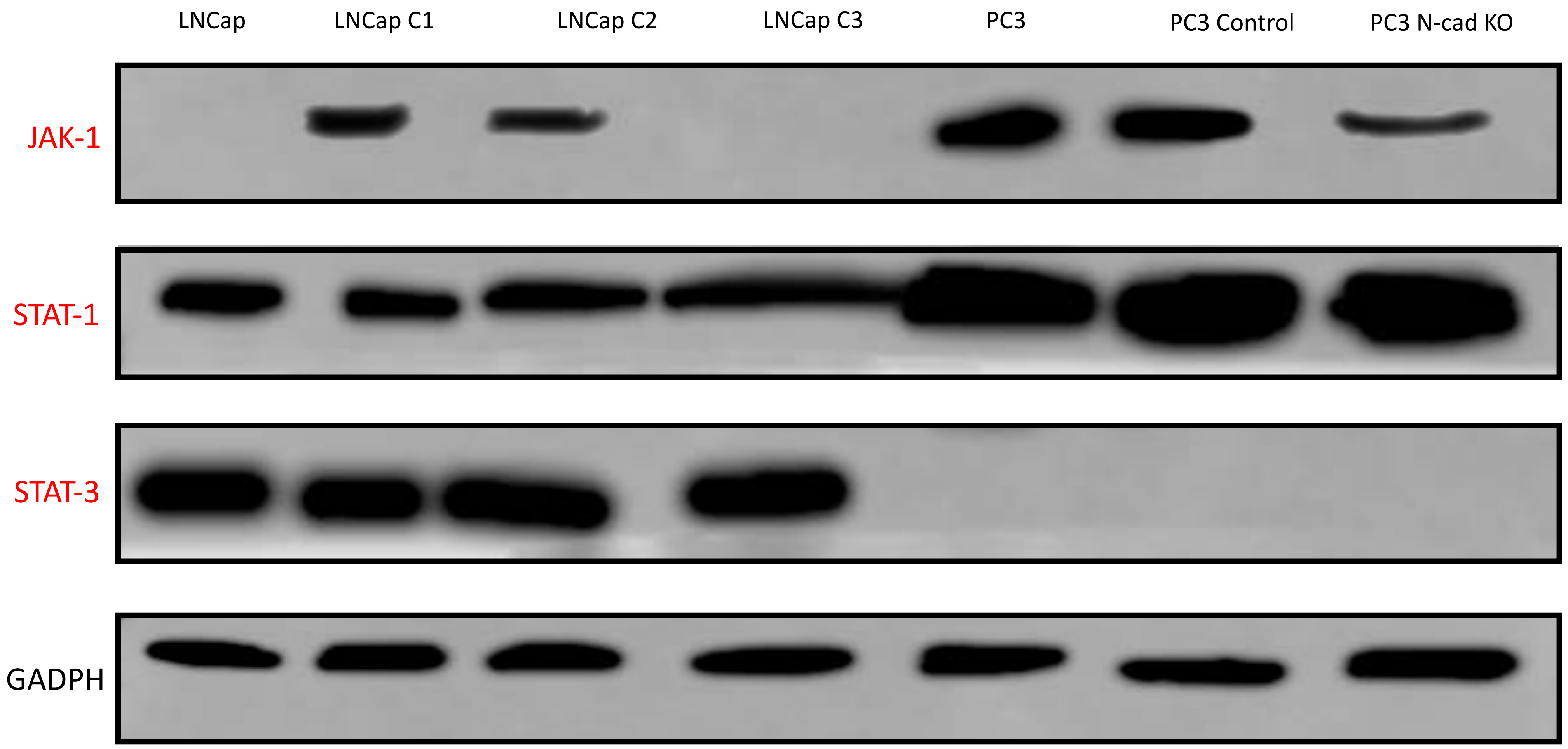
LNCap
LNCap C1
LNCap C2
LNCap C3
PC3
PC3 Control
PC3 N-cad KO
JAK-1
STAT-1
STAT-3
GADPH

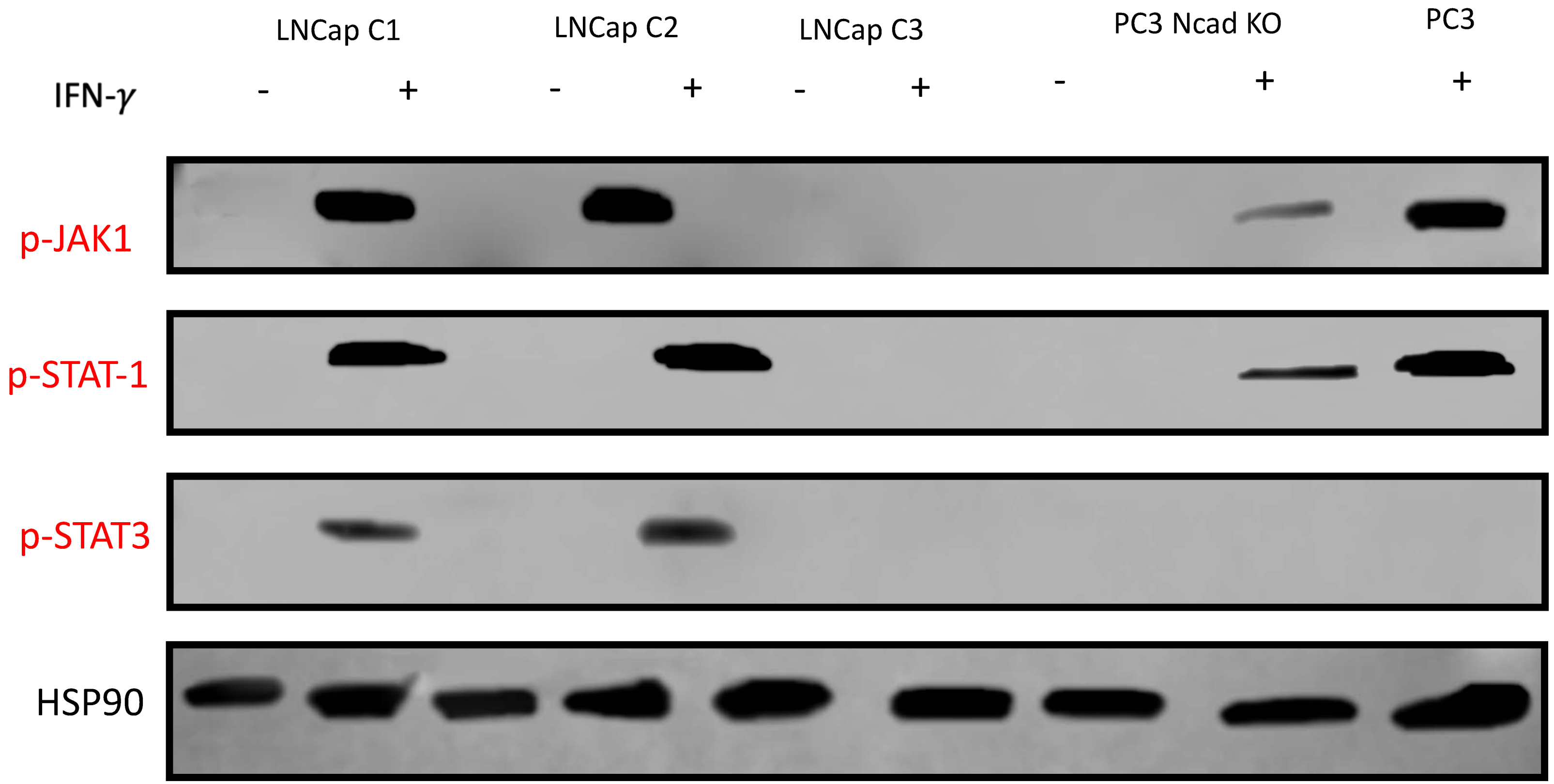
LNCap C1
LNCap C2
LNCap C3
PC3 Ncad KO
PC3
IFN-γ
- + - + - + - + +
p-JAK1
p-STAT-1
p-STAT3
HSP90

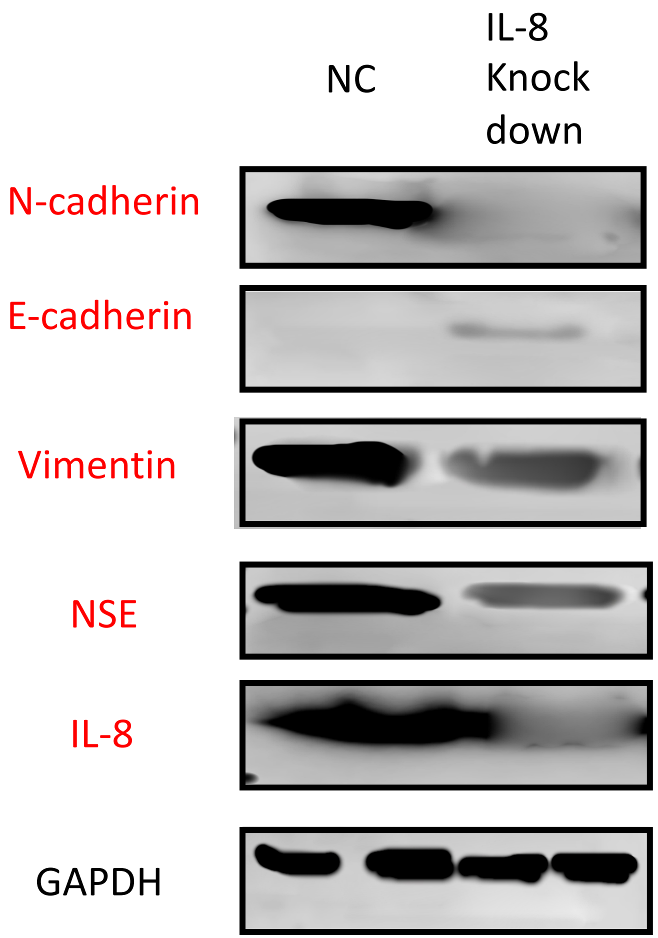
NC
IL-8 Knock down
N-cadherin
E-cadherin
Vimentin
NSE
IL-8
GAPDH

BMJ Publishing Group Limited (BMJ) disclaims all liability and responsibility arising from any reliance placed on this supplemental material which has been supplied by the author(s)

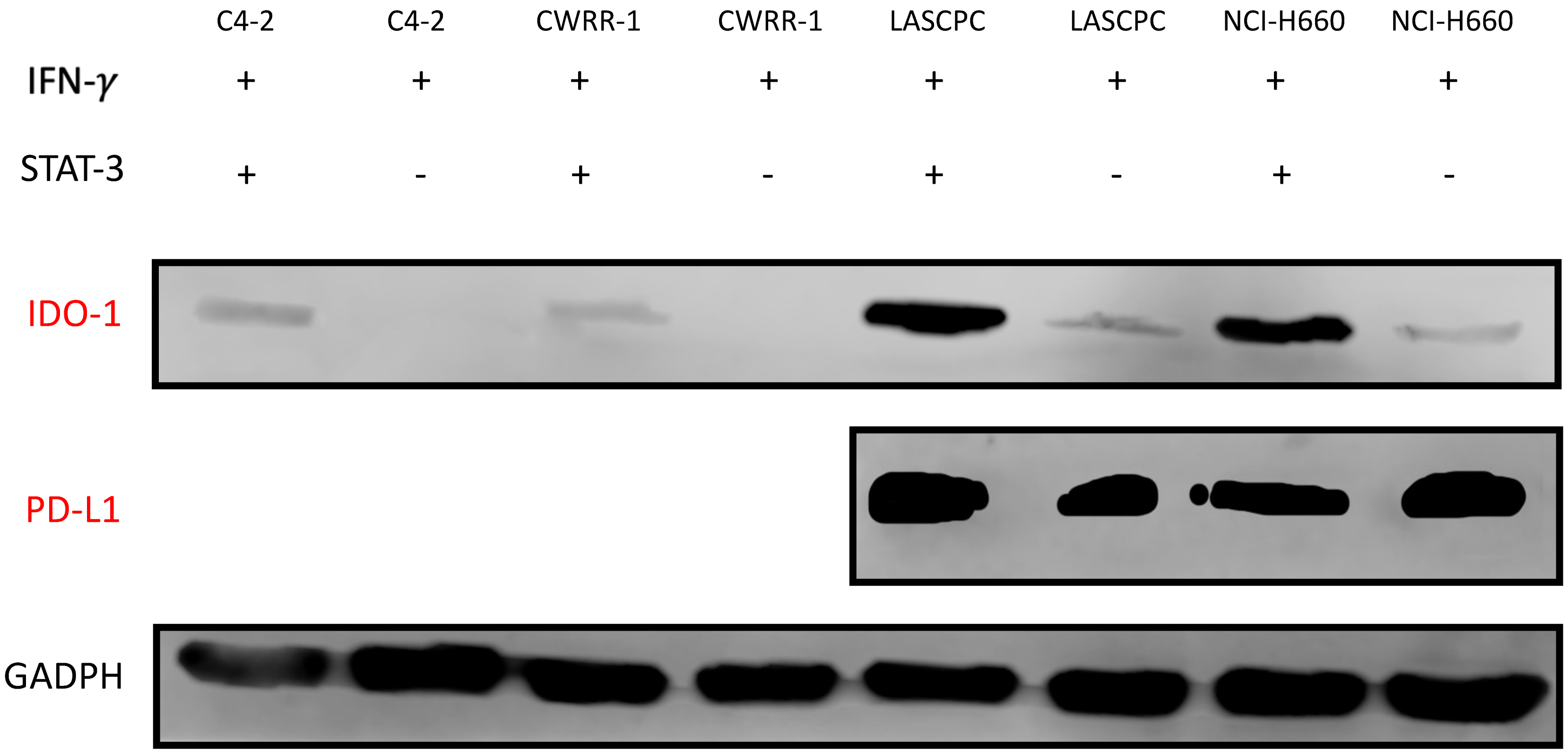

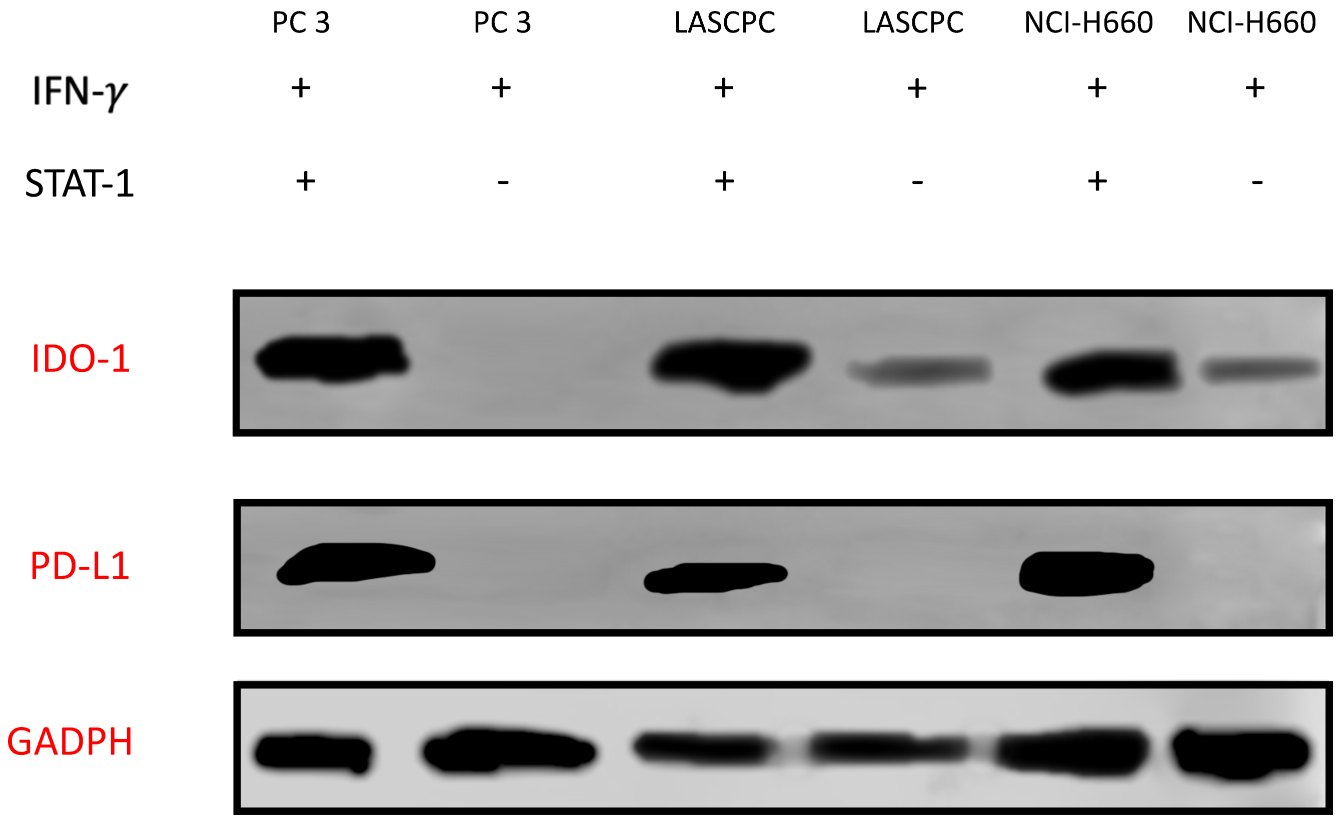
PC 3
PC 3
LASCPC
LASCPC
NCI-H660
NCI-H660
IFN-γ
+ + + + + +
STAT-1
+ - + - + -
IDO-1
PD-L1
GADPH

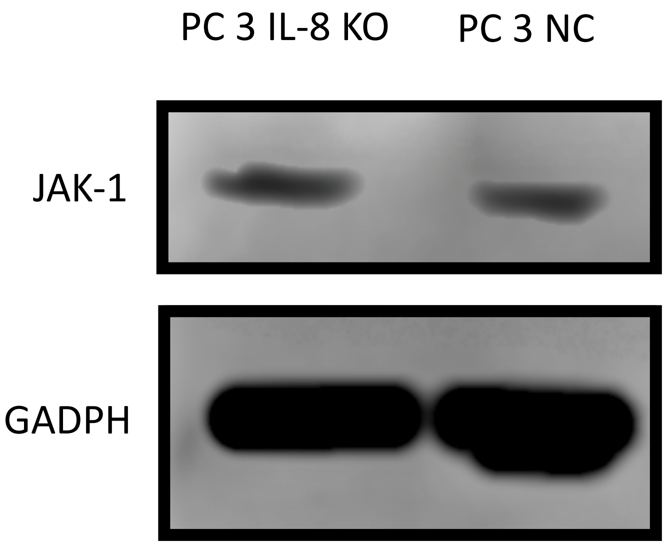
PC 3 IL-8 KO
PC 3 NC
JAK-1
GADPH

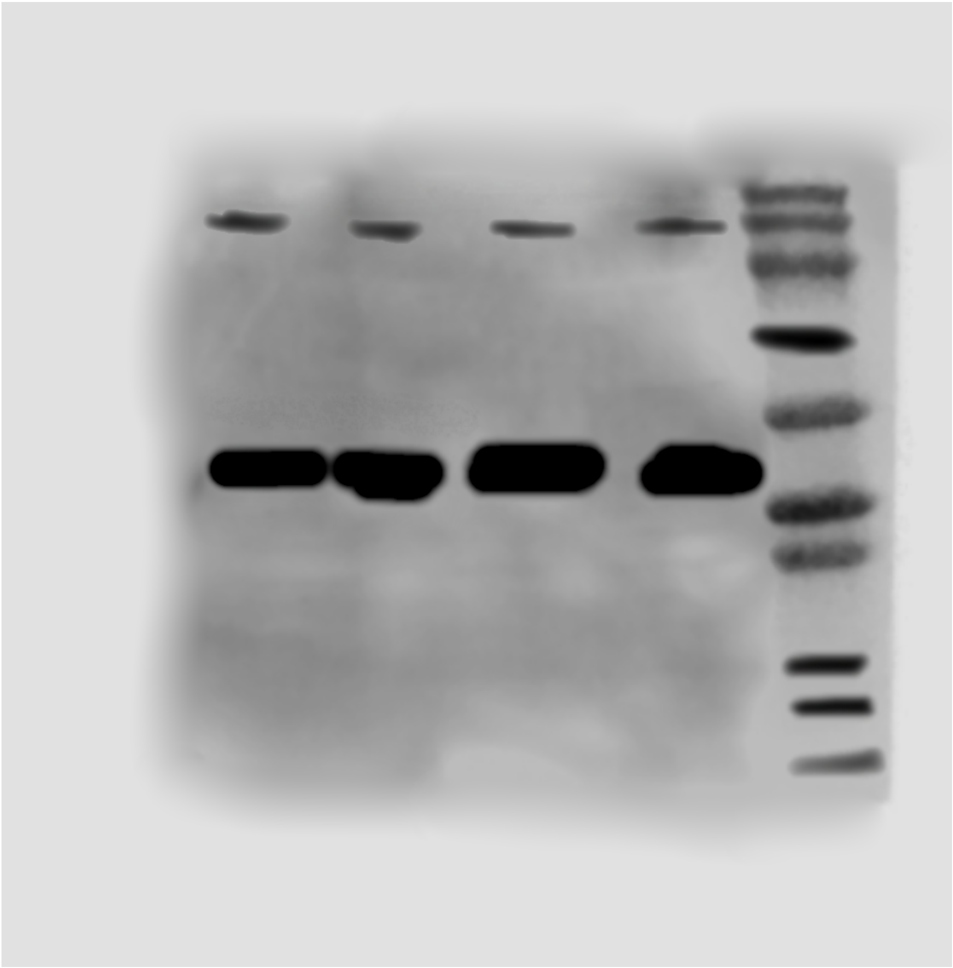

BMJ Publishing Group Limited (BMJ) disclaims all liability and responsibility arising from any reliance placed on this supplemental material which has been supplied by the author(s)

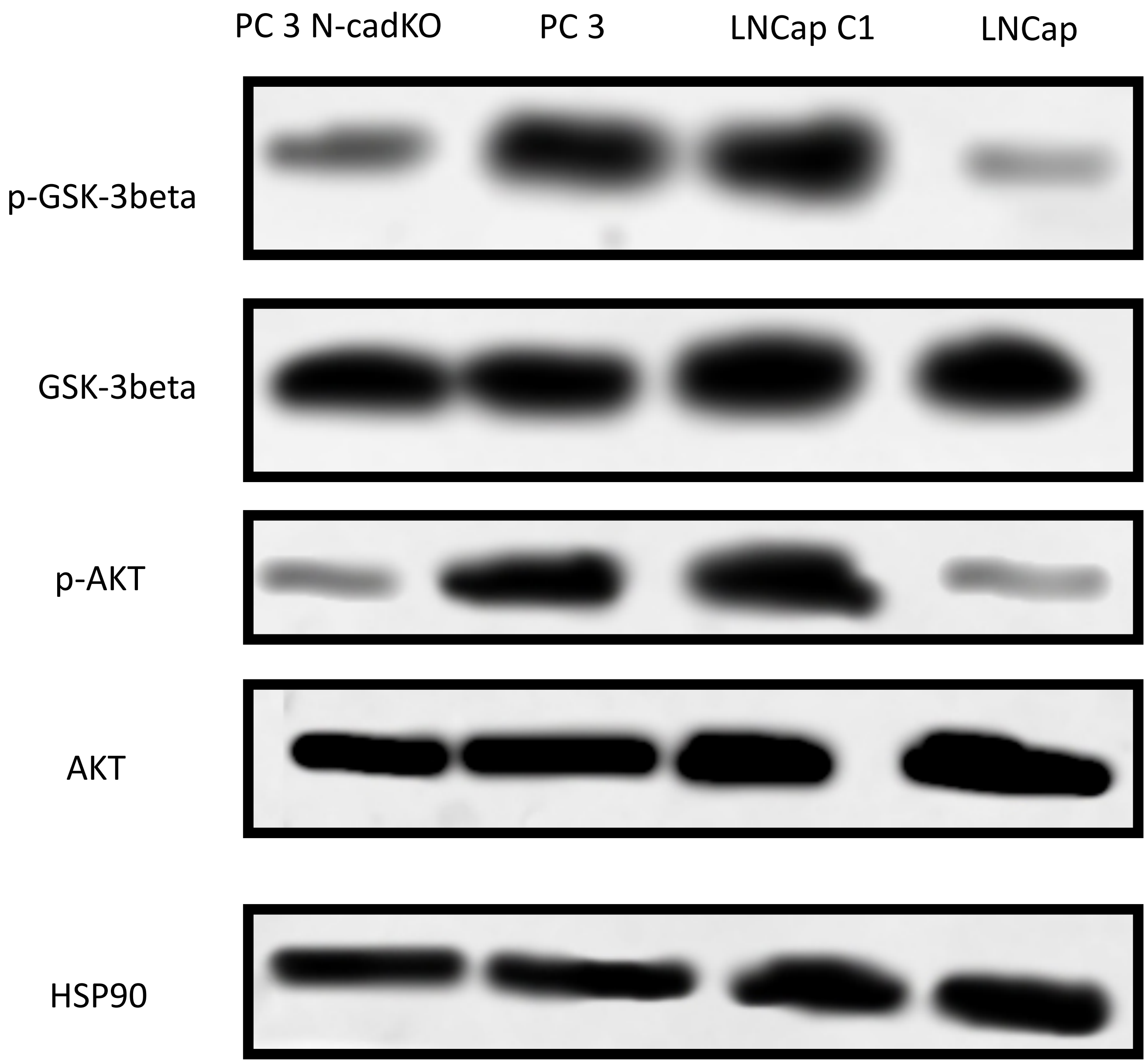

BMJ Publishing Group Limited (BMJ) disclaims all liability and responsibility arising from any reliance placed on this supplemental material which has been supplied by the author(s)

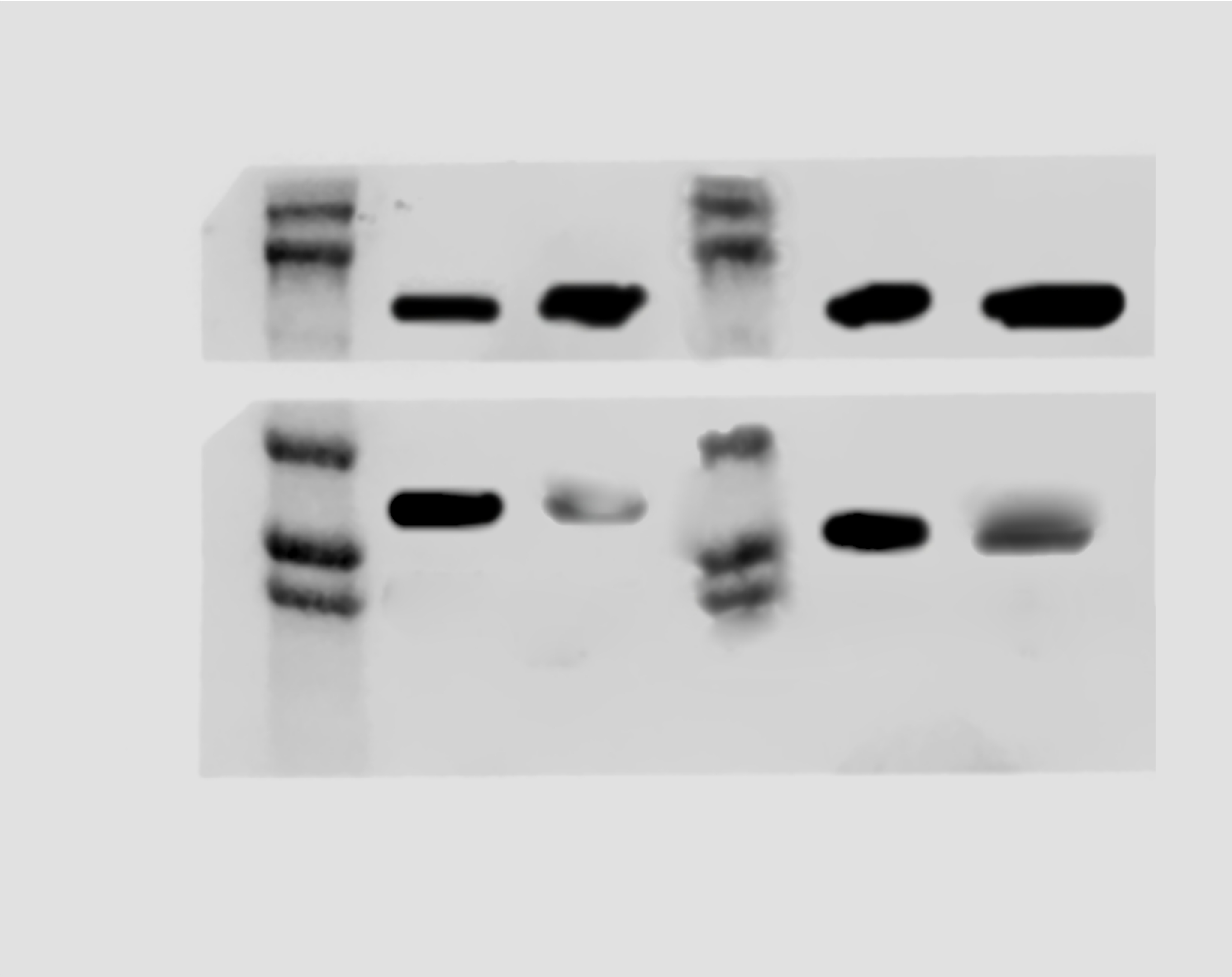

BMJ Publishing Group Limited (BMJ) disclaims all liability and responsibility arising from any reliance placed on this supplemental material which has been supplied by the author(s)

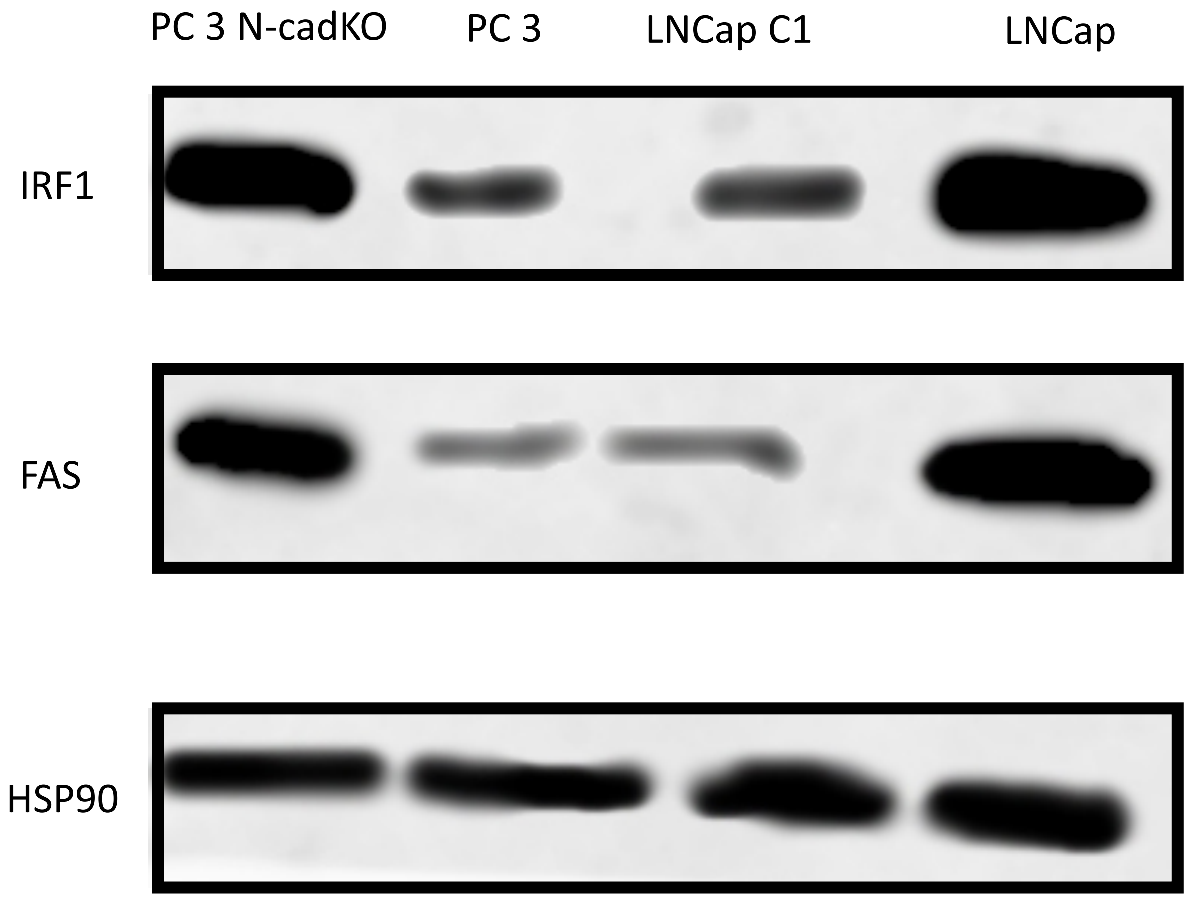

BMJ Publishing Group Limited (BMJ) disclaims all liability and responsibility arising from any reliance placed on this supplemental material which has been supplied by the author(s)

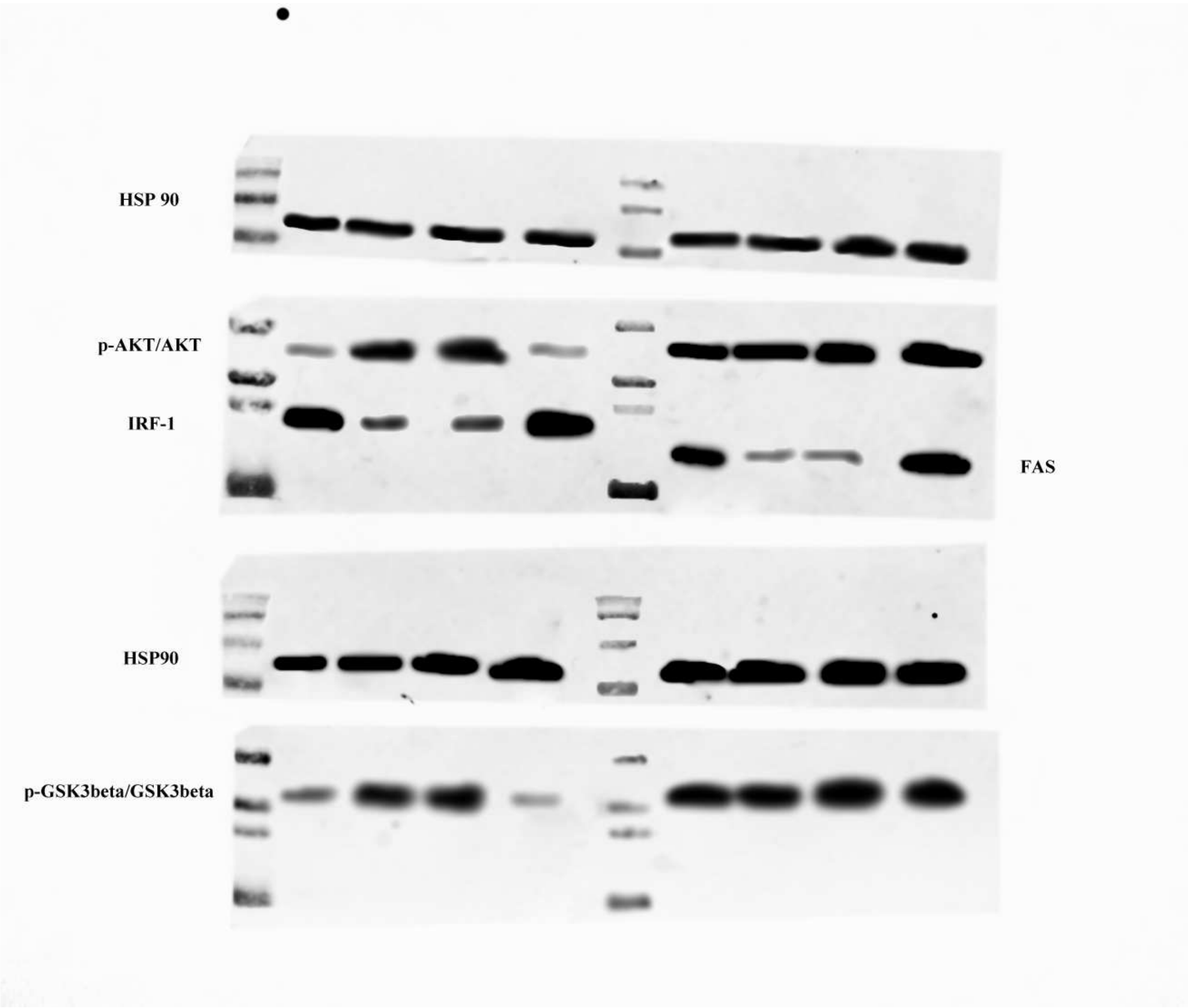

Supplement: Supplementary data [file jitc-2020-002138supp001.pdf]
