# Supplementary material for: N-cadherin inhibitor creates a microenvironment that protect TILs from immune checkpoints and Treg cells
Source: J Immunother Cancer. 2021 Mar 10;9(3):e002138. doi: 10.1136/jitc-2020-002138 (PMC7949480; doi:10.1136/jitc-2020-002138)
Supplement: Supplementary data [file jitc-2020-002138supp002.pdf]

Primers

IFNGR1:
forward, 5’-TCCTCAGTGCCTACACCAACTAATG-3’;

reverse, 5’-CTGGATCTCACTTCCGTTCATTCTC-3’.

JAK1:
forward, 5′-AATGTTCTCTATGAGGTCATGGTGACT-3′;

reverse, 5′-CAGTTTTTTCCGCTTCAGTTTATTT-3′.

STAT1:
forward, 5′ - GCTGGGCGTCTATCCTGTGGT- 3′;

reverse, 5′ - GCTCAGCTGGTCTGCGTTCA- 3′.

PD-L1:
forward, 5′-GGCATTTGCTGAACGCAT-3′;

reverse, 5′- CAATTAGTGCAGCCAGGT-3.

IDO-1:
forward, 5’-TCACAGACCACAAGTCACAGC-3’;

reverse, 5’-AGTTGGCAGTAAGGAACAGCA-3’.

CXCL 10:

forward, 5’- GAAATTATTCCTGCAAGCCAATTT -3’;

reverse, 5’- TCACCCTTCTTTTTCATTGTAGCA -3’.

CXCL 11:

forward, 5’- CAGTTGTTCAAGGCTTCCCC -3’;

reverse, 5’-TGCCACTTTCACTGCTTTTACC -3’.

IRF1:

forward, 5’- CCTGATACCTTCTCTGATGGACTCA -3’;

reverse, 5’- TGCATGTAGCCTGGAACTGTGT -3’.

FASN:

forward, 5’- CCAAGCAGGCACACACG -3’;

reverse, 5’- GGAGTGAATCTGGGTTGATGC -3’.
