# Supplementary material for: N-cadherin inhibitor creates a microenvironment that protect TILs from immune checkpoints and Treg cells
Source: J Immunother Cancer. 2021 Mar 10;9(3):e002138. doi: 10.1136/jitc-2020-002138 (PMC7949480; doi:10.1136/jitc-2020-002138)
Supplement: Supplementary data [file jitc-2020-002138supp003.pdf]

Supplementary Methods and Materials

IHC scores

The immunohistochemical staining results were assigned as the mean score considering both the intensity of staining and the proportion of tumour cells with an unequivocal positive reaction. Each section was independently assessed by two pathologists without prior knowledge of the patient data. A proportional rate of positive reactions was observed, and positivity was defined as the presence of brown signals in the cell cytoplasm. This measure is also known as the frequency, defined as follows: 0, less than 5%; 1, 5%-25%, 2, 26%-50%; 3, 51%-75%, 4, greater than 75%. However, for PD-L1, we used different criteria, where more than 1% was considered positive; thus, the scores were defined as follows: 0, less than 1%; 1, 1%-5%; 2, 6%-25%, 3, 26%-50%, 4, 51%-75%; 5, greater than 75%. When the staining was heterogeneous, each component was scored independently and summed for the results. For example, a specimen containing 75% tumour cells with moderate intensity (3×2=6) and another with 25% tumour cells with weak intensity (1×1=1) received a final score of 6+1=7.

Assay measuring the FFA concentration

To quantify the FFA concentration in culture medium, all cell lines were maintained in FBS-free RPMI medium supplemented with 10% lipids. Plasma was isolated from blood, and interstitial fluids from tumours were collected by centrifugation. The concentrations of total FFAs or glucose were assessed with the Free Fatty Acid Quantification Kit (BioVision, Zurich, Switzerland). LC-MS analysis was performed to determine the FFA species concentrations in culture medium. LC was performed with an LC-20ADXR ternary pump system equipped with a DGU-20A5R degassing unit, an SIL-20AC autosampler, and a CTO-20AC column oven (Shimadzu Co., Ltd., Kyoto, Japan). The LC system was coupled with an LTQ Orbitrap XL hybrid linear ion trap-Fourier transform mass spectrometer (Thermo Fisher Scientific, Waltham, MA, USA). FFAs were detected by obtaining the extracted ion chromatograms of the deprotonated ions ([M-H]-) at a mass tolerance of 10 ppm. Instrument control, data acquisition, and data processing were performed using Xcalibur 2.1.0 software (Thermo Fisher Scientific).

Antibodies

The antibodies used for both IHC and western blotting were as follows: anti-IDO-1 (CST-51851, Danvers, MA, USA); anti- IFN-γ receptor 1 (IFNGR1; Invitrogen A5-27841, Waltham, MA, USA); anti-p-IFNGR1 (Invitrogen PA5-38504, Waltham, MA, USA); JAK1 (Invitrogen PA5-86682, Waltham, MA, USA); p-JAK1 (Invitrogen Tyr1022, Waltham, MA, USA); and anti-N-cad (Thermo Fisher MA1-91128 CH-19, Waltham, MA, USA). The PD-L1 antibody used for western blotting and flow cytometry was CST-13684, and the antibody used for IHC was Dako Phe19-Thr239. The secondary antibody used for flow cytometry was conjugated to Alexa Fluor 488. Western blotting for STAT1, p-STAT1, STAT3 p-STAT3, E-cadherin and Vimentin was performed using the respective following antibodies: Santa Cruz (CA, USA) SC-464; Invitrogen KIKSI0803, Santa Cruz SC-8019 (CA, USA); CST-9145, Sigma SAB2104222; and Sigma V6389. IHC for IFN-γ was performed with the A259336 antibody purchased from Invitrogen. The PCR primers are shown in the Supplementary Materials. Western blotting antibodies of GSK-3β, p-GSK-3β, AKT, p-AKT, IRF1, FAS are from ThermoFish: C 367.3, Tyr 216, G 145.7, 14-6, SR 44-08, DX 2.
